# Transposable Element (TE) insertion predictions from RNAseq inputs and TE impact on RNA splicing and gene expression in *Drosophila* brain transcriptomes

**DOI:** 10.1186/s13100-024-00330-z

**Published:** 2024-10-09

**Authors:** Md Fakhrul Azad, Tong Tong, Nelson C. Lau

**Affiliations:** 1 Department of Biochemistry and Cell Biology, Boston University Chobanian & Avedisian School of Medicine, Boston, MA 02118 USA; 2 Graduate Program in Bioinformatics, Boston University, Boston, MA 02118 USA; 3 Genome Science Institute, Boston University Chobanian & Avedisian School of Medicine, Boston, MA 02118 USA

**Keywords:** Transposable element, Intron splicing, *Drosophila* brain, TE cis regulation

## Abstract

Recent studies have suggested that Transposable Elements (TEs) residing in introns frequently splice into and alter primary gene-coding transcripts. To re-examine the exonization frequency of TEs into protein-coding gene transcripts, we re-analyzed a *Drosophila* neuron circadian rhythm RNAseq dataset and a deep long RNA fly midbrain RNAseq dataset using our Transposon Insertion and Depletion Analyzer (TIDAL) program. Our TIDAL results were able to predict several TE insertions from RNAseq data that were consistent with previous published studies. However, we also uncovered many discrepancies in TE-exonization calls, such as reads that mainly support intron retention of the TE and little support for chimeric mRNA spliced to the TE. We then deployed rigorous genomic DNA-PCR (gDNA-PCR) and RT-PCR procedures on TE-mRNA fusion candidates to see how many of bioinformatics predictions could be validated. By testing a *w1118* strain from which the deeper long RNAseq data was derived and comparing to an *OreR* strain, only 9 of 23 TIDAL candidates (< 40%) could be validated as a novel TE insertion by gDNA-PCR, indicating that deeper study is needed when using RNAseq data as inputs into current TE-insertion prediction programs. Of these validated calls, our RT-PCR results only supported TE-intron retention. Lastly, in the *Dscam2* and *Bx* genes of the *w1118* strain that contained intronic TEs, gene expression was 23 times higher than the *OreR* genes lacking the TEs. This study's validation approach indicates that chimeric TE-mRNAs are infrequent and cautions that more optimization is required in bioinformatics programs to call TE insertions using RNAseq datasets.

**Supplementary Information:**

The online version contains supplementary material available at 10.1186/s13100-024-00330-z.

## Introduction

Transposable Elements (TEs) are insertional mutagens making up major fractions of animal genomes, yet we are still determining how TE mobilization affects gene expression, chromatin accessibility, and physiology. In multicellular organisms, most genes are filled with introns that are spliced out; thus, introns are generally safe harbors for TEs to insert into without overtly disrupting the protein-coding exons. Since the spliceosome can effectively and precisely splice out introns varying widely in size, the impact of TEs on gene regulation is hard to define if the TE sequences are invisible to the splicing machinery. If an intronic TE includes a sequence that can trigger alternative splicing, this can fuse the original gene exons to TE sequences to result in an ‘exonization’ event that incorporates TEs into novel mRNA isoforms [1, 2]. However, the extent of TE exonization in organism is still unclear because the field lacks extensive molecular validation of genomics predictions of chimeric TE-mRNA fusion transcripts arising from the revolution in high-throughput DNA and RNA sequencing.

To see if we could confirm intronic TEs splicing into host mRNA, we first considered a null hypothesis that perhaps most TEs residing in introns are not being exonized but rather acting as passive sequence platforms, which include evolving a selectable function for the binding of a transcription factor that connects the host gene to a new regulatory network [2–4]. A competing hypothesis is that TE exonization is frequent, and this proposition was recently examined by three independent studies [5–7] that devised custom bioinformatics programs to search transcriptome datasets from a lab stock and wild natural collections of *Drosophila melanogaster*.

The first study [7] developed a custom bioinformatics pipeline called TE-chim that uses the STAR aligner [8] to screen for sequencing reads that span the gene-TE junction and then predicts the TE insertion site in the corresponding genomic locus using BLAST [9]. This study posited that mRNA splicing to TEs to generate chimeric transcripts was a frequent event in the midbrain of the *Drosophila w1118* strain. A second study claimed that 19% of the body-part specific transcripts are gene-TE chimeras, and that on average, gene-TE chimeras can contribute up to 43% of the total gene expression in *Drosophila* [5]. A third study focusing on RNAseq datasets from *Drosophila* ovaries had a more conservative measure of ~ 1% of transcripts displaying such TE-gene chimeras [6].

In all three studies claiming TEs are frequently expressed as parts of chimeric mRNAs with genes, there was minimal experimental support beyond the RNA sequencing datasets. Some RT-PCR amplicons were shown in Oliveira et al. [6] without amplicon sequencing confirmation or gDNA-PCR validation. The Coronado-Zamora and Gonzalez study [5] was primarily based on bioinformatics findings and lacked PCR validation. Because these two studies primarily utilized wild *Drosophila* collections to highlight the diversity of TE landscapes and differential contribution of TEs to nearby genes, the ability to distribute and reproduce these natural *Drosophila* isolates is restricted. The Treiber and Waddell study [7] did not have any additional gDNA-PCR and RT-PCR experiments to back up the bioinformatics predictions from the RNAseq data from a *w1118* strain derivative.

Our understanding of how intronic TEs impact a gene’s transcript maturation remains incomplete because standard DNAseq and RNAseq analysis algorithms are optimized to map sequencing reads to a reference genome and transcriptome, whereas intronic TEs that are either novel or even in a reference will not usually be included in gene models. Therefore, we developed our own bioinformatics algorithms for analyzing deep sequencing data for novel TE insertions and we have shown that TE landscapes are extremely diverse amongst *Drosophila* strains [10]. Our Transposon Insertion and Depletion AnaLyzer (TIDAL) program was originally designed around Whole Genome Sequencing (WGS) DNAseq datasets as inputs and is tuned to maximize specificity over sensitivity [10].

However, the next frontier would be to examine how TIDAL can handle RNAseq data as an input to primarily search for TE insertions in transcribed regions of the genome. Since RNAseq datasets are much more numerous and diverse from model organisms to human clinical samples, there could be a good potential to leverage bioinformatics analysis of RNAseq data to discover novel TE landscape. Additionally, we hypothesize that TIDAL could also be a valuable benchmarking tool to examine the extent of TE-gene exonization events in *Drosophila* transcriptomes. Thus, in this study we applied TIDAL to two *Drosophila* head and brain RNAseq datasets [7, 11] that have exceptionally deep RNA sequencing coverage that would not present a limitation compared to WGS.

Since *w1118* is one of the most widely used *Drosophila* lab strains, our study here applies rigorous experiments to validate TE-gene chimeric events predicted by bioinformatics programs. Although TE landscapes are known to be diverse among *Drosophila* strains, our study uses gDNA PCR to see which TE insertions are commonly shared between the *w1118* strain of our study and the previous study. Then we applied RT-PCR on those common TE insertions near mRNAs to assess how frequently TE-gene chimeras accumulate compared to the standard gene transcripts. Our careful approach to TE-insertion validation shows that TE-gene chimeric transcripts are still very low in frequency amongst *Drosophila* brain/head transcriptomes. While there is potential to utilize TIDAL to find novel TE insertions from RNAseq data, our program and others need to contend with unappreciated artifacts from RNAseq data that can reduce the confidence in valid TE insertion calls.

## Results

### Considering RNAseq data as input for TIDAL TE insertion predictions

**Fig. 1:**
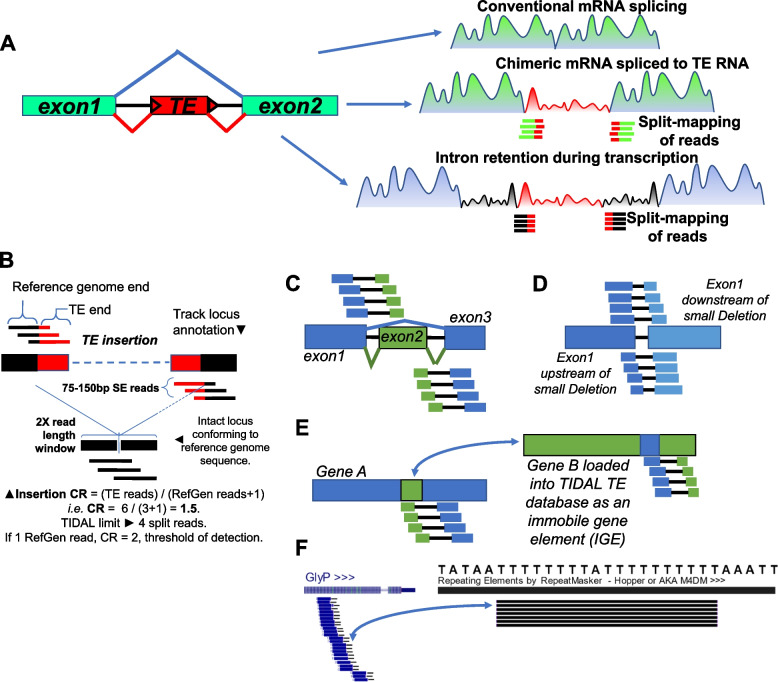
The chimeric TE-mRNA concept and TIDAL implementation using RNAseq data as input. **A **Diagrams considering how read coverage would reflect canonical exon splicing versus a TE-gene chimera versus intron retention during transcription of the intronic TE. Split reads representing these de novo TE insertions would not be mapped to a reference genome and transcriptome, requiring a specialized bioinformatics program like TIDAL and others. **B**-**E **Diagrams of TIDAL implemented on RNAseq to detect TEs in (**B**), Alternative splicing isoforms (**C**), small deletions like InDels (**D**), and potential gene-fusions that are more likely artifacts of similarity in sequences between different genes because some genes are loaded into TIDAL as an IGE (immobile gene element) (**E**). Depicted are split reads being aligned to the genomic structural variant by scripts within TIDAL. **F **A common artifact of a simple T-repeat sequence from reverse-transcribing from the Poly-A during Alternative Poly-Adenylation (APA) of a *Drosophila* gene like *GlyP*, where this simple T-repeat is part of the *hopper/M4DM* TE sequence. This additional simple-polynucleotide filter was added to TIDAL runs on RNAseq data

If intronic TEs were commonly exonized, we would envision two scenarios for how the RNAseq read coverage would diverge from canonical intron splicing (Fig. 1A). If the TE sequence is seamlessly spliced to a gene’s exons, there should be RNAseq reads that span a seamless junction between the exon to the TE sequence. Alternatively, there could be transcription from the host gene promoter or autonomous transcription initiation from promoters within the intronic TE, and this scenario of intron retention would be reflected by RNAseq reads that span the intron-TE junctions.

We introduce these concepts because TIDAL and other de novo TE insertion prediction programs [10, 12–16] use split-mapping of RNAseq reads where one end must map to unique sequence in the reference genome and the other end must map to a database of TE consensus sequences. In the TIDAL algorithm design, split-mapping of DNAseq reads follows the regimen portrayed in Fig. 1B [10]. In a later version of TIDAL [17], we added a similar control feature of genes added into the repeats database to act as “Immobile Genetic Elements” (IGEs) as described in [7]. These IGEs help measure a false prediction rate in TIDAL that was below 12% in WGS DNAseq [17].

However, when we examined *Drosophila* head RNAseq data as input into TIDAL, the excessive number of IGEs being flagged in TIDAL outputs revealed a complexity in split-mapping of RNAseq reads that was not apparent in DNAseq reads. We will discuss the frequencies and fractional proportions of these IGE idiosyncrasies further below, but in Fig. 1C–F we first introduce these idiosyncrasies as diagrammed concepts. Because TIDAL splits a read to map each end within a window size (Fig. 1B), many IGEs can trick a TIDAL call if the reads span across the splicing of tiny introns (i.e. < 100nt, Fig. 1C, Supplemental Figure S1) and genomic structural variants (SVs) like small insertions and deletions (InDels, Fig. 1D, S1A).

If a WGS library is properly prepared with complete shearing and sampling of the entire genome, the sequence diversity is immense and thoroughly distributed across the multitude of reads. Transcriptomes, however, may only represent < 10% of the entire sequences of an animal’s genome, with different expression levels that can bias many gene sequences over others, and different protein-coding genes can share short similar sequences if they are encoding a commonly shared protein domain. To fully consider RNA maturation steps, we conjecture that short sequence compositions within RNAseq inputs are influencing the significant differences in TIDAL outputs compared to WGS DNAseq inputs.

Furthermore, the input of RNAseq into TIDAL also raised the calls of two distinct genes that might appear to form a two-mRNA fusion transcript (Fig. 1E), whereas these types of calls were low with DNAseq inputs. The TIDAL program [10] was originally designed to take as input WGS DNAseq from Illumina reads as short as 50 nucleotides (nt), and uses the Bowtie v1 algorithm [18] for split-mapping of each read’s end using just 22nt but allowing up to 3 mismatches. Given how transcriptome sequences are just more naturally biased in short sequences that could be similar between genes compared to entire genomes (Fig. S1B), the frequency of finding these kinds of shared alignments between disparate genes became quite high. We favored this interpretation over the other molecular possibility of artifactual mis-priming of distinct gene amplicons during PCR amplification steps of RNAseq library construction [19, 20].

Lastly, this short read-alignment mismatch problem resulted in TIDAL detecting an abnormally high number of putative insertions of the *hopper*/*M4DM* TE in genes (Fig. 1F). In the two-gene fusion issue illustrated in Fig. S1B, we noticed a pattern of a simple sequence like Poly-T in the *Zelda* gene, this could have been generated from reverse-transcription of a Poly-A tail during library preparation. There is also a Poly-T sequence within the *hopper/M4DM* TE sequence that TIDAL was latching onto to calling an excessive number of false positives. WGS DNAseq libraries are immune from this Poly-T artifact that is easy to see being formed in RNAseq libraries. For these RNAseq inputs, we added filtering steps within TIDAL to remove the Poly-T artifact and cut down on false *hopper/M4DM* calls. However, other simple shared sequences between two distinct genes were still too numerous and diverse for us to develop a suitable filter to screen away other two-gene artifacts. We are still studying the annotation information within TIDAL output tables to find better ways to spot these two-gene artifacts in the future.

### TE-mRNA fusion calls are a minority of TIDAL prediction events from *Drosophila* neurons and brain RNAseq inputs

We first tested TIDAL’s functionality on RNAseq by inputting high-quality transcriptomes from four purified sets of *Drosophila* neurons repeatedly sampled across 6 circadian time points [11]. These 48 RNAseq libraries enabled us to examine how reproducible or variable were potential TE-mRNA fusion calls by TIDAL since these neurons should have at least a large backdrop of consistently expressed genes with a subset of circadian oscillating genes. We also tested a very-deep longer-read (250 × 250PE) RNAseq dataset from the Treiber and Waddell study that proposed frequent TE exonization events and non-autonomous TE transcript expression in *Drosophila* midbrains [7].

**Fig. 2:**
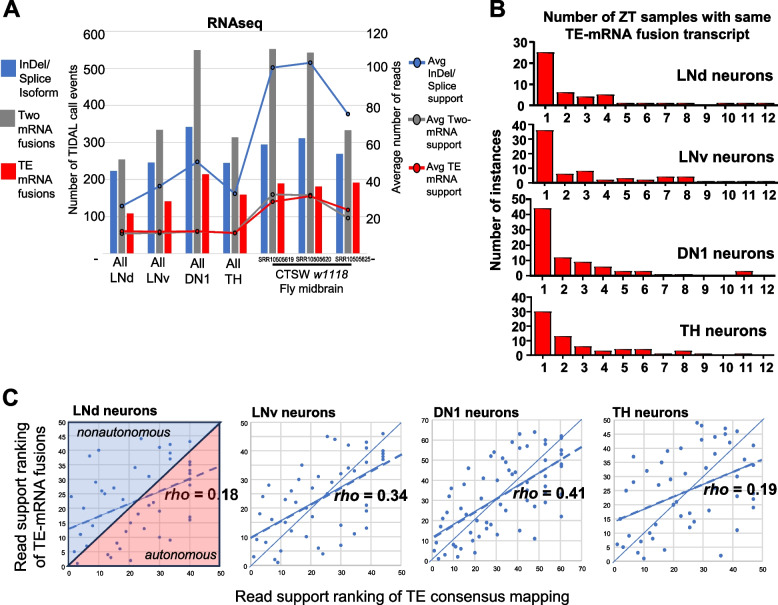
Comparing TE-mRNA fusions and insertion calls versus other TIDAL calls from *Drosophila* brain RNAseq inputs. **A **Histograms of the number of the Zeitgeiber Time (ZT) samples supporting the same consistent TE-mRNA fusion calls by TIDAL in the circadian rhythm RNAseq dataset. **B **How RNAseq inputs perform in TIDAL with the bar graph showing the number of TIDAL calls (left Y-axis number) for events representing an InDel/Splice isoform, a two-mRNA fusion call, or a TE-mRNA fusion call. Lines in the bar graph display the average number of reads (Right Y-axis) that support the different TIDAL calls. **C **Scatterplots show some positive correlation between read support ranks of TEs in TE-mRNA fusions versus consensus sequence mapping events. The diagonal-splitting lines of each scatterplot separates the non-autonomous TEs expression tied to the gene in the upper-diagonal half versus the autonomous TE expression dots that are in the lower-diagonal half

We tracked the variation in library sequencing depths and read-mapping proportions to the *Drosophila* reference genome for each of the 48 circadian rhythm *Drosophila* neurons RNAseq libraries (Figure S2A). We also merged each of the 12 libraries into their single neuron type (LNd, LNv, DN1, and TH, Fig. S2B), and after analyzing all these RNAseq datasets as inputs into TIDAL, we then counted the TIDAL outputs by three categories: (1) TE-mRNA fusions as our main feature of interest, (2) a genuine molecular event of InDels and Splicing isoforms that activate a TIDAL call, and (3) artifacts of two mRNAs being called as a fusion event (Fig. 2A, Fig. S2A). Regardless of variations in library reads depth and genome-mappable read fractions in these circadian rhythm RNAseq datasets, two-mRNA fusions were the major proportion of TIDAL calls followed by InDel/Splicing isoform calls.

If TE-mRNA fusions were a common occurrence, then TIDAL should have found more reproducible examples with robust read support from such a large RNAseq dataset with this many time points and this many neuronally expressed genes being extensively profiled. Instead, TE-mRNA fusion calls were the minority, between 18–22% in these TIDAL calls.

These circadian neuron RNAseq libraries have 12 timepoints that can serve as replicates to ask how many times a given TE-mRNA fusion call is seen repeatedly, which would lend support that the call is a true positive. The frequency histograms show that the vast majority of the TE-mRNA fusion calls are only seen in a single timepoint sample in this circadian RNAseq dataset (Fig. 2B). An independent RNAseq dataset from the *Drosophila* midbrain that was run through TIDAL also exhibited just 18–24% of the calls representing TE-mRNA fusion events, while two-mRNA artifacts also dominated in these TIDAL calls. Although TIDAL outputs enable simple detection and filtering of the false-positive Two-mRNA fusion events, we realize that TE-mRNA fusion events need to be inspected further.

A previous study claimed that all TE transcripts were expressed non-autonomously because TE expression would be largely defined by the host genes’ expression in the *Drosophila* brain RNAseq from outputs of their TEchim program [7]. However, the transformations used to compare TE transcripts to the host mRNAs were not clear, so we re-examined this claim with a more appropriate correlation analysis that compares the rankings of TE read counts in the consensus elements versus the TE-mRNA fusion calls (Fig. 2C), which does not use any arbitrary transformation and can yield a better comparison than Fig. 6A in the Treiber and Waddell study [7].

Overall, there was consistent positive correlation of TE-mRNA fusion call read support and TE-consensus coverage read support, as well as several TEs in our analysis that were in the upper-diagonal half of these scatterplots, which are consistent with non-autonomous TE expression as depicted in the scatterplot in Fig. 6A in the Treiber and Waddell study [7]. However, in contrast to the claim that all TE expression was non-autonomous, our analysis showed many TEs were expressed autonomously from the mRNA (lower-diagonal half), and this would explain the relatively low (< 42%) *rho* correlation coefficients. Our results indicate that TE-mRNA fusion events need more scrutiny to their validity than what the bioinformatics predictions may indicate.

### TIDAL performance differences between RNAseq and WGS-DNAseq as inputs

**Fig. 3:**
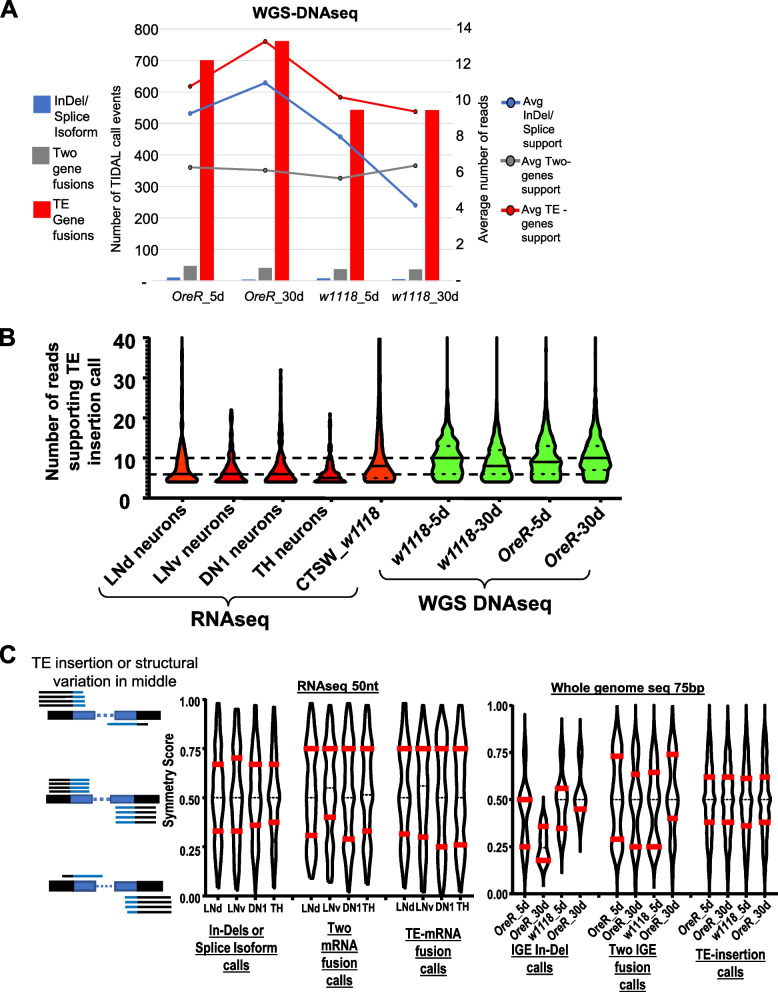
TIDAL performance differences between RNAseq and WGS-DNAseq as inputs. **A **Similar bar and lines graph as Fig. 2A but for WGS-DNAseq as the standard input into TIDAL. The TE- gene fusions in the WGS-DNAseq is a much greater proportion even though there is a lower number of supporting reads. **B **Violin plots showing a more skewed bias in RNAseq inputs (colored red) of generally fewer split reads supporting each event call, whereas DNAseq inputs (colored green) have a more balanced distribution of more split reads supporting each event call. TIDAL requires at least 4 reads that span the structural variation breakpoint to make a call, so all of these violin plots have a wide base at 4 reads. **C **Violin plots of the distribution of symmetry scores for TIDAL calls of genome structural variants from *Drosophila* neuron RNAseq versus whole fly WGS libraries. Red bars mark quartiles, the dashed midline is the mean

Since TIDAL only calls TE-mRNA fusion events as a minority of the outputted predictions from RNAseq inputs, we compared a set of recent WGS-DNAseq libraries analyzed under the same version of TIDAL [17]. Despite an average of ~ 50% fewer reads for each TIDAL event call compared to the RNAseq inputs, the WGS-DNAseq inputs showed that the overwhelming majority of events were TE insertions into gene-proximal loci (Fig. 3A). As expected from the biochemistry of WGS-DNAseq library preparation, calls for InDels were miniscule. The major artifact of Two-mRNA fusions making up most of TIDAL calls from RNAseq inputs was many-fold lower in the WGS-DNAseq inputs. These results reaffirm the robustness of TIDAL’s TE-insertion call outputs when WGS-DNAseq is used as inputs.

TIDAL was originally designed on using WGS-DNAseq Illumina sequencing reads as inputs, and the specificity of choosing valid calls was tuned with several quality filters [10].These quality filters require: (1) a minimum of 4 split reads of support covering the TE insertion junction, (2) that the ends of the split reads have a BLAT score [21] of > 50% to catch some of the unmasked and unannotated simple-sequence repeats, and (3) that these reads are spread out within the defined window size of 2X times the read length (Fig. 1B). The distribution of these split reads on either side of the TE insertion also allows us to calculate a “symmetry score” for each TE insertion call, such as a 50% score that is ideal symmetry (half reads on each side of the insertion), and scores towards the extremes of 1% and 99% exhibit biased distribution of the insertion-spanning reads.

When we compared these quality filter scores of TE insertion calls between RNAseq and WGS DNAseq inputs, the violin plots showed that the majority of TE-mRNA fusion calls in RNAseq were skewed at the low 4-read minimum (Fig. 3B), with the mean at just 6 supporting reads. In contrast, the distribution of the number of supporting reads for WGS-DNAseq was more balanced with the mean of 10 supporting reads for TE-insertion calls. There was also an extended upward tail of varying numbers of reads supporting TE insertion calls, reflecting aspects of non-uniform read coverages in both RNAseq and WGS-DNAseq libraries that we currently do not yet fully understand.

To illuminate the performance differences between these two types of high-throughput sequencing inputs, we compared symmetry score distributions for each of the event calls made by TIDAL for RNAseq versus WGS-DNAseq (Fig. 3C). We expect InDels/Splicing events in RNAseq to be a robust molecular feature for mature RNA transcripts, and the violin plots of the symmetry scores for InDels/Splicing events fit an archetypal shape – a central bulged mean at 50% and the quartiles nestled closer to this central mean. This archetypal violin plot shape was also shared in the TE-insertion calls’ symmetry scores in the WGS-DNAseq outputs, which have strong confidence in the validity of these event predictions. The presumptive artifacts, such as the Two-mRNA fusions in both inputs displayed deviating violin plot shapes from archetypal shapes of InDels/Splicing events in RNAseq and TE-insertions in WGS-DNAseq, suggesting that this parameter and others may be useful to better screen out artifacts.

## PCR validation of predicted TE insertion in *Drosophila* genomes from RNAseq inputs

To test whether mRNA-TE chimeras are frequently present in *Drosophila* brain transcriptomes as proposed in previous studies [5–7], we developed rigorous gDNA-PCR and RT-PCR approaches to assay gDNA and total RNA from *Drosophila w1118* and *Oregon-R* (*OreR*) heads. We selected 19 predicted TE-gene insertions that both TIDAL and TEchim predicted from the same *w1118* mid-brain RNAseq inputs (Table 1). We also selected 3 cases and 4 cases only predicted by TEchim or only by TIDAL, respectively. We acknowledge that any flies obtained now would be at least 5 years past the original flies used for the original RNAseq [7], so the goal is to identify TE-gene insertions still retained in the *w1118* flies for further experimental analysis. The summary of our gDNA-PCR results is tabulated in Table 1 and will be elaborated upon in the text discussion below.

**Table 1:**
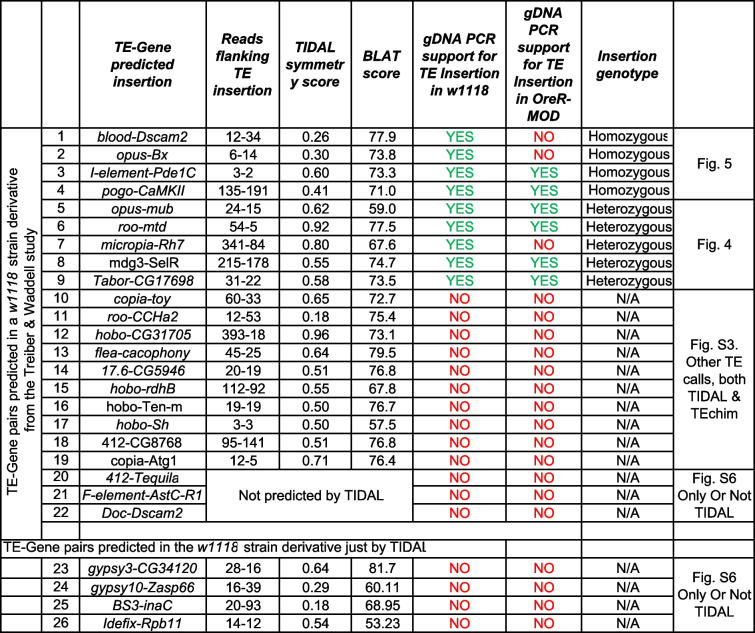
List of TE-Gene pairs evaluated by PCR in this study

**Fig. 4:**
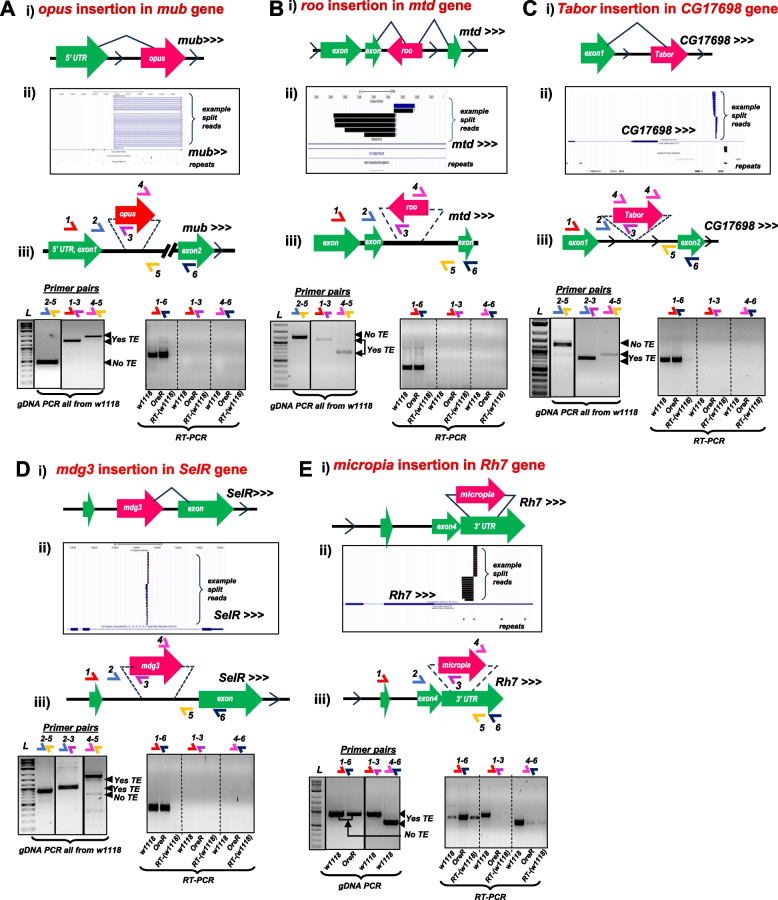
Rigorous gDNA-PCR validation of heterozygous TE insertions in selected genes in the *Drosophila w1118* strain genome. Panels correspond to the TE-gene pairs (**A**) *opus*-*mub,* (**B**) *roo*-*mtd,* (**C**) *Tabor-CG17698,* (**D**) *mdg3-SelR,* and (**E**) *micropia-Rh7.* Each figure panel is divided in parts (i) that is a diagram of the presumptive TE-gene splicing event proposed by Treiber and Waddell 2020, (ii) UCSC Genome Browser snapshots of the example split reads support for the TE insertion from TIDAL analysis of the *w1118* midbrain RNAseq data, (iii) gel images of gDNA-PCR amplicons (left set) and RT-PCR amplicons (right set) from the various sets of primer pairs illustrated in the diagram above the gel images. Solid lines around gels indicates cropped gel images; dashed lines represents different sections on a single gel

For each of these 26 cases, we conducted gDNA-PCR analysis with a series of primers to first validate if the predicted TE was indeed inserted into the predicted genomic regions of *w1118* and *OreR* strains. These panels consist of an amplicon called GPCR1 that are made by gene-specific primers that immediately flank the putative TE insertion, and amplicons GPCR2 and GPCR3 that use one primer that is gene-specific and the other primer binds to the adjacent terminus of the putative TE insertion (Supplemental Table S1, Fig. 4). When a TE insertion is true and too large for the GPCR1 primers to yield an amplicon (*i.e.* a full-length *opus* insertion is 7.5 kb [22], so GPCR1 primers 2–5 can only amplify the allele lacking the TE, Fig. 4A-iii), then the GPCR2 (primers 1–3) and GPCR3 (primers 4–5) amplicons are short enough to be efficiently amplified to confirm the TE inserted into the gene intron.

This experimental approach allowed us to confirm five TE insertions predicted by TEchim and TIDAL from RNAseq inputs, and our data show they are heterozygous in the *w1118* strain genomes (Fig. 4A-E). These TEs are *opus* insertion in the intron of *mub* (Fig. 4A), *roo* in the intron of *mtd* (Fig. 4B), *micropia* in the intron of *Rh7* (Fig. 4C), *Tabor* in the intron of *CG17698* (Fig. 4D), and *mdg3* inserted in a *SeIR* intron (Fig. 4E). We conclude that these TE insertions were heterozygous because the GPCR1 amplicon could only amplify the wild-type allele lacking the TE, whereas the GPCR2 and GPCR3 amplicons were detected and confirmed for the TE insertion by amplicon sequencing. Except for *Rh7-micropia*, all other heterozygous TE insertions were also observed in the *OreR* strain (Table 1, Supplemental Figure S3).

**Fig. 5:**
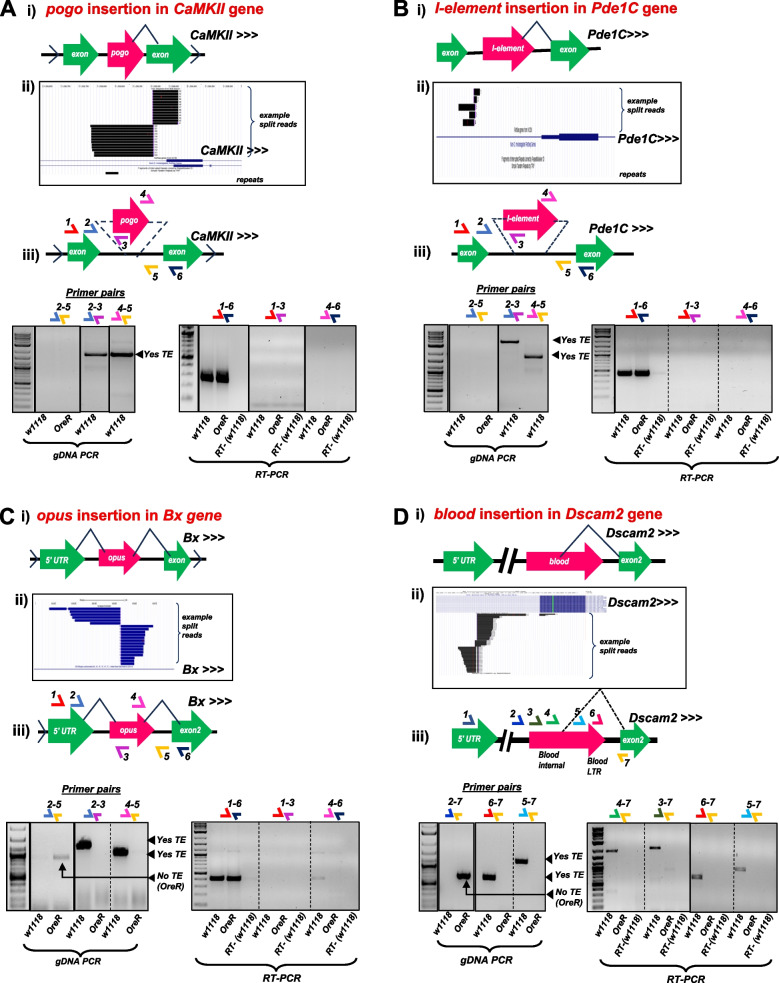
Rigorous gDNA-PCR validation of homozygous TE insertions in selected genes in the *Drosophila w1118* strain genome. Panels correspond to the TE-gene pairs (**A**) *pogo*-*CaMKII,* (**B**) *I-element-Pde1C,* (**C**) *opus-Bx,* and (**D**) *blood-Dscam2.* Each figure panel is divided in parts (i) that is a diagram of the presumptive TE-gene splicing event proposed by Treiber and Waddell 2020, (ii) UCSC Genome Browser snapshots of the example split reads support for the TE insertion from TIDAL analysis of the *w1118* midbrain RNAseq data, (iii) gel images of gDNA-PCR amplicons (left set) and RT-PCR amplicons (right set) from the various sets of primer pairs illustrated in the diagram above the gel images. Solid lines around gels indicates cropped gel images; dashed lines represents different sections on a single gel

With four other TE insertions also predicted by TEchim and TIDAL from RNAseq inputs, our data showed these were homozygous TE insertions in the *w1118* strain genomes (Fig. 5A-D). We confirmed a *pogo* insertion in the *CaMKII* gene intron (Fig. 5A), *I-element* in the *Pde1C* gene intron (Fig. 5B), *opus* insertion in the *Bx* gene intron (Fig. 5C), and *blood* insertion in the *Dscam2* gene intron in *w1118* flies (Fig. 5D). These TE insertions were homozygous in the *w1118* genome because a theoretical amplicon for GPCR1 primers was too long to be efficiently amplified thus was not detected in the gel, but GPCR2 and GPCR3 amplicons were amplified and confirmed by amplicon sequencing for TE insertion identity. Two of these homozygous TE insertions in *w1118* were also present and homozygous in *OreR* (Table 1, Fig. S3).

We interpret that these six TE insertions commonly shared between *w1118* and *OreR* strains were in the ancestral *D. melanogaster* strain, yet it is mysterious how five of these TEs can be maintained as heterozygous alleles when *Drosophila* transvection and meiotic recombination have not driven the allelic conversion to the homozygous TE insertion state [23, 24]. Alternatively, our perception of heterozygosity could also be complicated by local Copy Number Variation (CNV) flux when sampling bulk genomic DNA from multiple cells in tissue, head or the whole fly. In other words, CNV flux represents some fraction of cells that develop a genomic alteration or abnormal copy number of a particular genomic fragment amongst the backdrop of normal cells with unchanged genomes. This CNV flux is reflected by the Coverage Ratio (CR) values from TIDAL’s output of TE insertions for the w1118 genome sequenced in our previous study [17], most TEs have high CR values indicative of homozygous TE insertions in *w1118,* but there are also significant numbers of TE insertions whose CR values are < 1.5 that is either a heterozygous TE or a local CNV flux (Supplemental Figure S4A)*.* Interestingly, global genomic CNV flux starts out very low in young 5-day old adult flies, but significantly increases in aged 30-day adults (Supp. Fig. S4B). Since we do not yet have experimental approaches to distinguish CNV flux from heterozygosity in the TE insertion, we maintain using the latter term for now, but other examples of genomic CNV flux and TE heterozygosity in *Drosophila* have also been described [25, 26].

Of the 17 remaining TE insertion predictions, our gDNA-PCR experiments were unable to confirm an actual TE insertion in the intron of the host gene in neither *w1118* nor *OreR* (Table 1). Although four and three putative TE insertions were only predicted by either TIDAL or TEchim, respectively, the other nine of these TE insertion predictions were predicted by both programs, and we noted the relatively high number of supporting split reads that appeared to flank the TE insertion and displayed ideal TIDAL symmetry scores and BLAT scores. The negative results of testing for TE insertions predicted by both TEchim and TIDAL are shown in (Supplemental Figure S5), while TE insertions only predicted by either TIDAL or TEchim are shown in (Supplemental Figure S6). Only the GPCR1 amplicon indicated a wild-type genotype at each gene intron location, with no GPCR2 or GPCR3 amplicons detected. These results indicate the majority (~ 65%) of the predicted TE insertions using RNAseq as inputs could potentially be false positives that will require deeper study because our current TIDAL metrics (high supporting split reads, symmetry scores, and BLAT scores) cannot yet distinguish between the 9 true-positive TE insertions from the 17 false-positive predictions.

## Effect of transposon on host gene RNA splicing and steady state mRNA accumulation

We utilized the similar primers that validated the 9 TE genomic insertions to examine for possible TE-mRNA chimera formation in an RT-PCR assay. In the rightmost subpanel of Fig. 4Aiii to 4Diii and Fig. 5Aiii to 5Ciii, the RT-PCR results only showed a robust amplicon corresponding to the complete canonical splicing of the two exons flanking the intron harboring the TE. Other primer pair amplicons designed to detect a potential TE-mRNA chimera repeatedly failed to detect a clear sign of the putative TE-mRNA chimera proposed by Treiber and Waddell [7]. Notably, all of the split reads in the RNAseq output from TIDAL TE insertion prediction are spanning the intronic RNA directly flanking the TE insertion. None of the split reads in the TIDAL outputs appeared to span a splicing junction between the TE and the host gene’s exons. This negative result is supported by our additional negative results of no distinct RT-PCR amplicons corresponding to the TE-mRNA chimera.

Only two TE insertions in genes yielded amplicons supporting a TE-mRNA chimera. There is a true TE-mRNA fusion of *micropia* inserted in the 3' UTR of the *Rh7* gene, but because this is an exon, splicing did not contribute to this TE-mRNA chimera. In addition, this TE does not affect the *Rh7* open reading frame but might impact the mRNA’s expression level (Fig. 4E)**.** The *blood* TE is inserted into the first intron of the *Dscam2* gene in the *w1118* strain (Fig. 5D), but contrary to the proposition by Treiber and Waddell [7] that *blood* was splicing frequently into the second exon, our RT-PCR results only detected transcription indicative of intron retention, not splicing (Fig. 5Diii). Sanger sequencing of the RT-PCR amplicon showed the small portion of the *Dscam2* intron was retained in the amplicon next to the *blood* sequence, whereas had TE-splicing been true, the amplicon sequencing would have lacked intronic sequence. This RT-PCR result is consistent with the TIDAL outputs showing the vast majority of TE split reads between *blood* and *Dscam2’s* first intron are only supporting intron retention. Between the gDNA-PCR and RT-PCR results, there is little experimental support for validating TE-mRNA chimeras that are predicted from the analysis using RNAseq inputs.

**Fig. 6:**
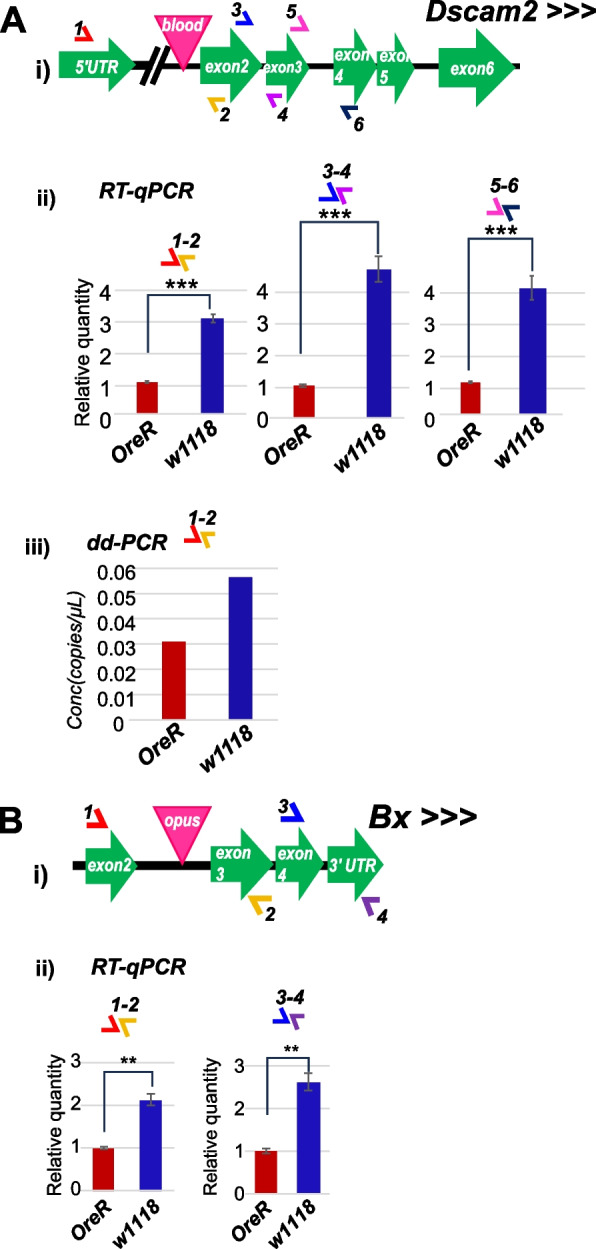
Testing the effect of a TE insertion within an intron on host gene mRNA splicing and expression. Diagrams showing the *w1118* strain has *blood* TE insertion in *Dscam2* gene (**A**-i) and *opus TE* insertion in *Bx* gene (**B**-i), while the *OreR* strain does not have those TE insertions in the corresponding genes. (**A**-ii) RT-qPCR assay examining spliced *Dscam2* mRNA levels in the heads of *w1118* and *OreR*. (**A**-iii) ddPCR assay replicating the RT-qPCR result in (A-ii). (B-ii) RT-qPCR assay examining spliced *Bx* mRNA levels in the heads of *w1118* and *OreR*. T-tests p-values: * = *p* < 0.05, ** = *p* < 0.005, *** = *p* < 0.001

Nevertheless, the polymorphic TE intronic insertions in the *Dscam2* and *Bx* genes in only the *w1118* strain but not in the *OreR* strain presented an interesting opportunity to test how a TE insertion would impact the efficiency of host mRNA splicing or steady level of mRNA accumulation. We designed a series of exon-specific primer pairs spanning the intron containing the TE in the *w1118* strain as well as additional exons separated by introns downstream (Fig. 6). We then quantitated amplicon amounts normalized relatively to the *Rp49* housekeeping gene and repeatedly observed 2-to-fourfold greater *Bx* and *Dscam2* transcript levels, respectively, in the *w1118* head RNAs compared to *OreR* fly heads (Fig. 6Aii and Bii). The relative quantitation methodology showing greater host gene mRNA expression in *w1118* compared to *OreR* was confirmed by an independent absolute quantitation methodology with droplet-digital PCR (ddPCR, Fig. 6Aiii).

If the *blood* and *opus* TEs inserted into *Dscam2* and *Bx* introns, respectively, are full length, they each would expand the intron sizes by ~ 7.5 kb. We could not disentangle if the TE insertion was counterintuitively enhancing intron splicing, or if the TE was serving as an enhancer to increase transcription activation because downstream exon amplicons were just as elevated in *w1118* versus *OreR*. Our results clearly show the absence of supporting evidence for alternative splicing that would generate TE-mRNA chimeras, but these two examples could represent the first cases of *Drosophila* TEs providing a regulatory element to enhance gene expression, similar to examples in mammals reviewed in [27, 28].

## Discussion

Although recent studies [5–7] have claimed that TEs in *Drosophila* may be frequently splicing into host gene exons to generate TE-mRNA chimeras, the interpretations have mainly relied upon bioinformatics predictions without introspection of the supporting read patterns and PCR validation. One study performed some RT-PCR experiments on potential TE-mRNA chimeras [6], yet there was no confirmation by gDNA-PCR nor utilization of multiple primer pairs to ensure experimental rigor. The extensive gDNA-PCR and RT-PCR analyses in our study demonstrate the lack of evidence supporting TE-mRNA chimeric splicing. Thus, the question remains unanswered: how extensively do TEs affect host gene expression, via splicing or perhaps other mechanisms like transcription activation via acting like an enhancer platform [29]?

In WGS data, TIDAL’s stringent filters use these cutoffs: (1) requiring split reads on both insertion junctions of a novel TE insertion; (2) prioritizing the symmetry of the split reads around the reference genome insertion site; (3) discarding split reads that still contained repetitive signatures from a low BLAT score; and (4) enabling the program to return all the split reads from a TE insertion call so that a user can apply an orthogonal query of the split reads on the *Drosophila* genome from the UCSC Genome Browser. We validated TIDAL’s specificity by testing 49 predicted TE-insertions that we could validate up to 88% of these events with gDNA-PCR [10, 30]. Although TIDAL does not put out as many candidate TE insertion predictions as other tools that promote higher sensitivity (i.e. [12–16]), we have greater confidence that TE insertions called in WGS by TIDAL can be confirmed by gDNA-PCR.

A transcriptome is a constrained subset of the genome’s entire sequence diversity, and this feature may be reflected in our study, whereby RNAseq as inputs into bioinformatics programs like TEchim and TIDAL can lead to the same artifact predictions of TE insertions that cannot be validated by gDNA-PCR (Table 1). Currently, TIDAL can faithfully detect TE insertion events using WGS-DNAseq data at a validation rate of > 66% [10], whereas using RNAseq inputs lowers the validation rates to ~ 40% (9 of 23 tested TE insertion calls). We are still updating TIDAL to enable it to search for indicative patterns that will help us screen out artifactual two-mRNA fusion events to improve the confidence in TE insertion calls and TE-mRNA chimeras. Another future goal will be to test TIDAL with input data from long-read RNA sequencing methods that may resolve inconsistencies in chimeric transcript calls.

By reanalyzing previously published RNAseq data and confirming TEs inserted into mRNA loci, our experiments with RT-PCR verification then could not find support for these TEs splicing into mRNAs [5–7]. Moreover, there is the unaddressed concern of imprecision with TE exonization, which would generate many deleterious and non-functional transcripts turned over by Nonsense Mediated Decay (NMD) processes [31]. From a few studies in plants [32, 33] to one notable study in human cells involving ORF0 sequence located in the 5'UTR of the LINE-1 retrotransposon [34], there can be instances where TE-mRNA chimeras could form, but these studies acknowledge that NMD likely safeguards most deleterious transcripts from impacting host organism fitness.

In addition, the RNA interference (RNAi) pathway also silences TE expression in *Drosophila*, and we demonstrated that augmenting RNAi improves longevity by mitigating negative effects of increased TE expression in aged flies [17], a phenotype that has also been frequently observed in mammals [35–38]. We favor the idea that RNAi is mainly silencing TE transcripts that are autonomously expressed (Fig. 2C) without also silencing the host genes where it inserted because TE-mRNA chimeras are infrequent.

We acknowledge that the literature includes examples of TEs inserting into host genes and affecting gene expression positively to yield potentially novel functions. In one case**,** successive insertion of the *Accord, HMS-Beagle,* and *P*-element TEs in the *Drosophila* cytochrome P450 (*Cyp6g1*) gene improved resistance to the insecticide DDT [39]**.** A second example is a *Doc* TE insertion in the coding sequence of *CHKov1* that truncates CHKov1 mRNA to encode a shorter peptide that confers greater sigma virus resistance to *Drosophila* [26]**.** A third case is the KRABINER fusion gene of a mariner TE and KRAB domain protein modulating gene expression in bat cell cultures [40], but its true biological function in the bat animal remains unclear.

Lastly, TEs may exert regulation in *cis* to host genes via serving as novel enhancer platforms that recruit new transcription factor binding situations [3, 4, 27, 29]. We are speculating this hypothesis could explain the influence of the intronic insertion of *blood* and *opus* TEs into *Dscam2* and *Bx* neuronal genes respectively, in just the *w1118* fly strains that shows a higher level of *Dscam2* and *Bx* mRNA expression compared to the control *OreR* that completely lack these TEs in the corresponding genes (Fig. 6). The functional consequence of these higher *Dscam2* and *Bx* mRNA levels in *w1118* flies compared to *OreR* is not yet clear, but future genetics and functional genomics analyses are needed to improve our understandings of TEs impact on gene expression and overall fitness of an organism.

## Materials and methods

### Accessing RNAseq data sets

For this analysis, we downloaded a publicly available RNAseq dataset of *Drosophila* circadian rhythm cycling neurons [11] from NCBI Accession #GSE77451. This time-series data set contains 48 RNA samples extracted from 4 types of neurons in fruit fly brains, including dorsal lateral neurons (LNds), ventral lateral neurons (LNvs), dorsal neurons group 1 (DN1s), and dopaminergic neurons (TH). We also downloaded the full RNAseq data from the *w1118 Drosophila* midbrain from the Treiber and Waddell study [7] under the NCBI Accession #PRJNA588978.

### Detecting TE insertions using TIDAL and TEchim

We first used the TIDAL program [10] to detect TE insertions for each sample and pooled samples for each type of neuron studied in Abruzzi et al. [11] and then in the *w1118 Drosophila* midbrain from the Treiber and Waddell study [7]. Specifically, TIDAL removed the adaptor sequences, Poly-A, and low-quality bases from raw reads and duplicates. Then, the pre-processed reads were aligned to the *Drosophila melanogaster* reference genome Release 6 (Dm6) and from there unmapped reads, which potentially contain de novo inserted TE segments, were kept. Viral RNA, structural RNA, Repbase sequence, and TE sequence were further removed.

Next, to identify the TE-gene junction, 22nt-long sequences at both 5' and 3' ends were taken from each read and mapped to the TE consensus sequence, immobile gene elements (IGE) database, and repeat-masked Dm6 reference genome. Split reads with one end mapped to TE or IGE and the other end uniquely mapped to the reference genome were kept. Then, the reads within the 300nt size range that has one end mapped to the same TE were clustered together and clusters with size larger than 4 reads were kept. To further reduce false positives, clusters with BLAT score > 83% and span size smaller than (read length / 2) – 22nt were filtered. A metric called Coverage Ratio, which is the ratio between the number of split reads containing TE and the number of Dm6 mapped reads plus a pseudo count, was also calculated for additional filtering [10]. We also implemented a screen to remove sequences against the *hopper* TE sequence.

TEchim [7] used a similar strategy to detect TE insertions. After pre-processing steps including splitting reads at both ends, TEchim mapped these in-silico paired-end reads to a repeat-masked reference genome combined with TE consensus sequence. Reads covering TE-gene breakpoints were kept as input for BLAST program for annotation. However, TEchim doesn’t have a step to remove adaptors like TIDAL has, so we used Trimmomatic software to remove adaptors from raw fastq files.

After running TIDAL and TEchim programs, we summarized unique TE insertions across all 12 samples for each type of neurons. Unlike TEchim, which was designed only on finding TE insertion, TIDAL can also identify gene translocation and insertion/deletion variants. Therefore, we counted the number of unique insertions based on the annotation of the inserted sequence and its neighboring gene. We only utilized our TEchim run to confirm the results from the previously published Treiber and Waddell study [7], and to confirm the reproduced predictions listed in Table 1 and Table S1.

### Genomic DNA extraction and PCR

The *D. melanogaster w1118-iso* and *OreR-MOD* strains were used in this study. Both strains were raised at 25°C on standard cornmeal food. Genomic DNA were extracted from 7-day old 30 female fly heads using NEB Monarch Genomic DNA Purification Kit following the manufacturer provided protocol. DNA quantity and quality was checked on NanoDrop One Microvolume UV–Vis Spectrophotometer (ThermoFisher).

Genomic PCR was performed using NEB Phusion High-Fidelity DNA Polymerase (GC buffer) with different primer combinations described in Table S2 with an input of approximately 50 ng/ul DNA. Genomic PCR1 (GPCR1) was performed using a gene- specific forward primer located upstream of the hypothesized TE insertion position within the gene and a gene specific reverse primer downstream of the TE insertion position. Genomic PCR2 (GPCR2) was performed using the same gene-specific forward primer used in the GPCR1 and a TE-specific reverse primer, whereas GPCR3 was performed using the gene specific reverse primer used in the GPCR1 and a TE specific forward primer. All primer sequences are listed in Table S2.

### RNA extraction, cDNA synthesis, RT-PCR, quantitative RT-PCR (RT-qPCR), and droplet digital PCR (dd-PCR)

Total RNA was extracted from 7 day old 30–50 female fly heads using NEB Monarch Total RNA Miniprep Kit and quantified using Thermo Fisher Scientific NanoDrop One Microvolume UV–Vis Spectrophotometer. First Strand cDNA synthesis was performed according to the NEB specified protocol using random primers, ProtoScript II (NEB), and 1 μg of total RNA input.

Quantitative PCR (qPCR) was performed using the NEB Luna Sybr-Green mastermix with different primer sequences described in Table S2. For different gene and primer combinations, qPCR reaction was optimized by making a serial dilution of the original cDNA reaction ranging from 2 to 10X dilution. Relative changes in gene expression were calculated using the 2^ΔΔCt method with *Rp49* as a housekeeping gene for normalization. Briefly, the ΔCt value difference between target gene (*Dscam2* and *Bx*) and housekeeping gene (*Rp49*) was calculated for *w1118* (experimental group-TE insertion) and *OreR-MOD* (control-no TE insertion); and the difference between these two ΔCt values (dΔCt experiment- dΔCt control) was further calculated to obtain the ΔΔCt value. Relative fold change values (from experiment to control) were calculated from the exponent of 2 to the power of negative ΔΔCt value.

Droplet digital PCR (ddPCR) was conducted according to the protocol described in Yang et al. [17]. Briefly, ddPCR was performed on a Bio-Rad QX200 instrument with the ddPCR Evagreen Supermix (Biorad). Copy number measurements for specific genes (Table S1) were normalized to *Rp49* using 2 ng of cDNA as input per 20 μL ddPCR for droplet generation. For genes with very high copy numbers (for example, *Rp49*) that saturate the droplets, input cDNA was diluted further into the ddPCR mix prior to droplet generation. At least 12,000–15,000 droplets were generated to achieve a good statistical estimation of the concentration calculated by Poisson distribution using Quantasoft Analysis Pro (Biorad).

## Supplementary Information

Supplementary Material 1: Figure S1. Examples of why TIDAL is calling events like gene InDels/Splicing events and false two-mRNA fusion events from using RNAseq reads as inputs. (A) UCSC Genome browser coverage plots of RNAseq reads from the *w1118* *Drosophila *midbrain of (i) a small InDel in the *IA-2* gene and (ii) intron-spanning reads of the *PNUTS* gene, which are genes loaded into TIDAL as “Immobile Genetic Elements” (IGEs).  TIDAL seemed to flag these reads as a false SV call.  (B) Browser plots and gene sequence snapshots demonstrate that when short split ends of longer RNAseq reads were mapped by TIDAL, sequences are commonly shared between *Rbp9* and *elav* (i) and *kdn *with another simple repeat of Poly-T’s in *Zelda* appear to cause false positive gene “fusions” being called by TIDAL.

Supplementary Material 2: Figure S2. Supporting analyses of *Drosophila* head/brain RNAseq and DNAseq inputs into TIDAL from results in Figures 2 and 3. (A) Stacked bar and line graphs tallying the number of TE-mRNA fusion transcripts across the different ZT samples (library identifier in parentheses) in the different *Drosophila* neuronal types. TIDAL also reports deletion segments within the 100-control mRNA-coding genes and fusions between another mRNA with the control mRNA-coding gene. Note the two sets of legends corresponding to the different left and right Y-scale axis. The right Y-scale shows the number of total and uniquely-mapping reads as well as number of unmapped reads. (B) Read mapping statistics of the total RNAseq data for each *Drosophila* neuron type where all Zeitgeiber timed samples were merged and then subjected to TIDAL analysis.  (C) Read mapping statistics of *OreR* and *w1118* wild-type whole genome DNA sequencing for comparison to RNAseq depths.

Supplementary Material 3: Figure S3. Rigorous gDNA-PCR tests that confirm no TE insertion in the *Drosophila* *OreR *strain genome. Each figure panel is divided in parts (i) that is a diagram of the presumptive TE-gene splicing event proposed by Treiber and Waddell 2020, (ii) gel images of gDNA-PCR amplicons from the various sets of primer pairs illustrated in the diagram above the gel images. Solid lines around gels marked cropped gel images; dashed lines are demarcating lane sections on a single gel.

Supplementary Material 4: Figure S4. WGS-DNAseq analyzed by TIDAL finds TE insertions that can exhibit signatures indicative of a “heterozygous” state, and somatic DNA copy number variation can shift and be more sporadic as flies age. (A) Box plot of the Coverage Ratio scores for all the TE insertions called by TIDAL from WGS-DNAseq libraries of w1118 flies from two adult ages, from Yang et al 2022.  (B) Snapshots of the Chromosome 2L from the *w1118* flies from the Yang et al 2022 study, Copy Number Variation computed by the CONTROL-FREEC program shows that as flies age, the somatic genome copy number can start to fluctuate, and these fluctuations may also contribute to the varying Coverage Ratios in measuring TE insertions from WGS-DNAseq.

Supplementary Material 5: Figure S5. Rigorous gDNA-PCR tests that cannot validate predicted TE insertions in the *Drosophila* *w1118 *strain genome. Panels correspond to the TE-gene pairs (A) *hobo-Ten-m,* (B)* hobo-Sh,* (C)* 17.6-CG5946,* (D) *hobo-rdhB., *(E) *copia-toy,* (F) *roo-CCHa2, *(G) *412-CG8768, *(H)* hobo-CG31705, *(I)* copia-Atg1, and *(J)* flea-cac.* Each figure panel is divided in parts (i) that is a diagram of the presumptive TE-gene splicing event proposed by Treiber and Waddell 2020, (ii) UCSC Genome Browser snapshots of the example split reads support for the TE insertion from TIDAL analysis of the *w1118* midbrain RNAseq data, (iii) gel images of gDNA-PCR amplicons from the various sets of primer pairs illustrated in the diagram above the gel images. Solid lines around gels marked cropped gel images; dashed lines are demarcating lane sections on a single gel.

Supplementary Material 6: Figure S6. Rigorous gDNA-PCR tests that cannot validate TE insertions predicted only by TIDAL or only by TEchim in the *Drosophila w1118 *strain genome. Panels correspond to the TE-gene pairs only predicted by TIDAL for (A)* gypsy10-Zasp66* *,* (B)* Idefix-Rpb11,* (C)* gypsy3-CG34120* *,* and (D) *BS3-inaC* Each figure panel is divided in parts (i) that is a diagram of the presumptive TE-gene splicing event  (ii) UCSC Genome Browser snapshots of the example split reads support for the TE insertion from TIDAL analysis of the *w1118* midbrain RNAseq data, (iii) gel images of gDNA-PCR amplicons from the various sets of primer pairs illustrated in the diagram above the gel images. Panels correspond to the TE-gene pairs only predicted by TEchim for (E)* F-element-AstC-R1* *,* (F)* Doc-Dscam2, and* (G)* 412-Tequila. *Each figure panel is divided in parts (i) that is a diagram of the presumptive TE-gene splicing event proposed by Treiber and Waddell 2020, (ii) UCSC Genome Browser snapshots of the example split reads support for the TE insertion from TIDAL analysis of the *w1118* midbrain RNAseq data, (iii) gel images of gDNA and RT-PCR (only G) amplicons from the various sets of primer pairs illustrated in the diagram above the gel images.

Supplementary Material 7: List of Gene TE pairs evaluated by PCR in this study with detailed genomic coordinates.

Supplementary Material 8: List of oligonucleotides used in this study.

## Data Availability

All of the primary data are contained in the manuscript.  Fly strains can be requested by contacting the corresponding author.

## Data availability

No datasets were generated or analysed during the current study.
