# Supplementary material for: Transposable Element (TE) insertion predictions from RNAseq inputs and TE impact on RNA splicing and gene expression in Drosophila brain transcriptomes
Source: Mob DNA. 2024 Oct 9;15:20. doi: 10.1186/s13100-024-00330-z (PMC11462757; doi:10.1186/s13100-024-00330-z)
Supplement: Supplementary file 1 — Supplementary Material 1: Figure S1. Examples of why TIDAL is calling events like gene InDels/Splicing events and false two-mRNA fusion events from using RNAseq reads as inputs. (A) UCSC Genome browser coverage plots of RNAseq reads from the w1118 Drosophila midbrain of (i) a small InDel in the IA-2 gene and (ii) intron-spanning reads of the PNUTS gene, which are genes loaded into TIDAL as “Immobile Genetic Elements” (IGEs). TIDAL seemed to flag these reads as a false SV call. (B) Browser plots and gene sequence snapshots demonstrate that when short split ends of longer RNAseq reads were mapped by TIDAL, sequences are commonly shared between Rbp9 and elav (i) and kdn with another simple repeat of Poly-T’s in Zelda appear to cause false positive gene “fusions” being called by TIDAL. [file 13100_2024_330_MOESM1_ESM.pdf]

Azad, Tong et al.
Figure S1.

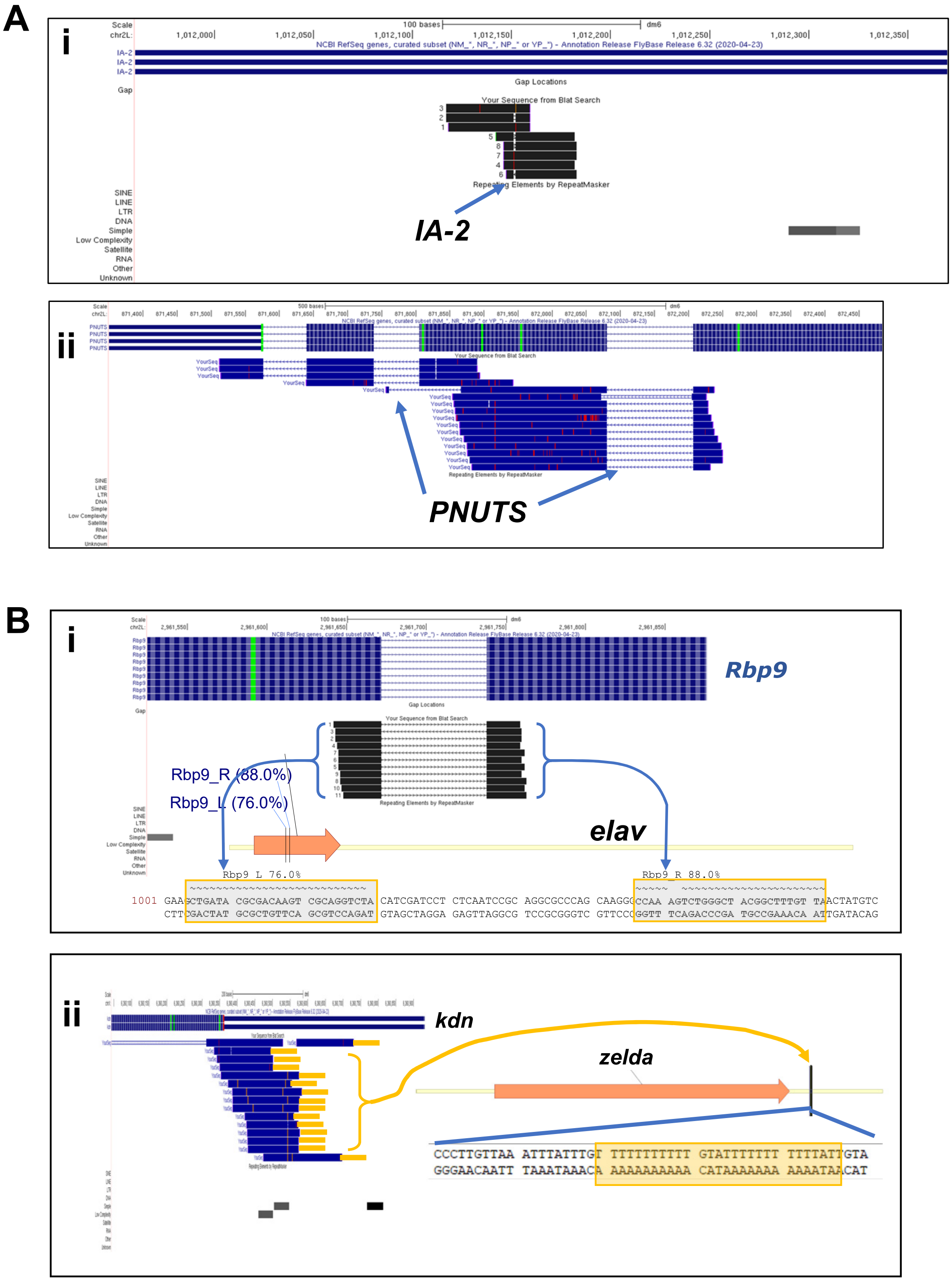

Figure S1. Examples of why TIDAL is calling events like gene InDels/Splicing events and false two-mRNA fusion events from using RNAseq reads as inputs.
(A) UCSC Genome browser coverage plots of RNAseq reads from the *w1118 Drosophila* midbrain of (i) a small InDel in the *IA-2* gene and (ii) intron-spanning reads of the *PNUTS* gene, which are genes loaded into TIDAL as "Immobile Genetic Elements" (IGEs). TIDAL seemed to flag these reads as a false SV call. (B) Browser plots and gene sequence snapshots demonstrate that when short split ends of longer RNAseq reads were mapped by TIDAL, sequences are commonly shared between *Rbp9* and *elav* (i) and *kdn* with another simple repeat of Poly-T's in *Zelda* appear to cause false positive gene "fusions" being called by TIDAL.
