# Supplementary material for: Transposable Element (TE) insertion predictions from RNAseq inputs and TE impact on RNA splicing and gene expression in Drosophila brain transcriptomes
Source: Mob DNA. 2024 Oct 9;15:20. doi: 10.1186/s13100-024-00330-z (PMC11462757; doi:10.1186/s13100-024-00330-z)
Supplement: Supplementary file 2 — Supplementary Material 2: Figure S2. Supporting analyses of Drosophila head/brain RNAseq and DNAseq inputs into TIDAL from results in Figures 2 and 3. (A) Stacked bar and line graphs tallying the number of TE-mRNA fusion transcripts across the different ZT samples (library identifier in parentheses) in the different Drosophila neuronal types. TIDAL also reports deletion segments within the 100-control mRNA-coding genes and fusions between another mRNA with the control mRNA-coding gene. Note the two sets of legends corresponding to the different left and right Y-scale axis. The right Y-scale shows the number of total and uniquely-mapping reads as well as number of unmapped reads. (B) Read mapping statistics of the total RNAseq data for each Drosophila neuron type where all Zeitgeiber timed samples were merged and then subjected to TIDAL analysis. (C) Read mapping statistics of OreR and w1118 wild-type whole genome DNA sequencing for comparison to RNAseq depths. [file 13100_2024_330_MOESM2_ESM.pdf]

Azad, Tong et al.
Figure S2.

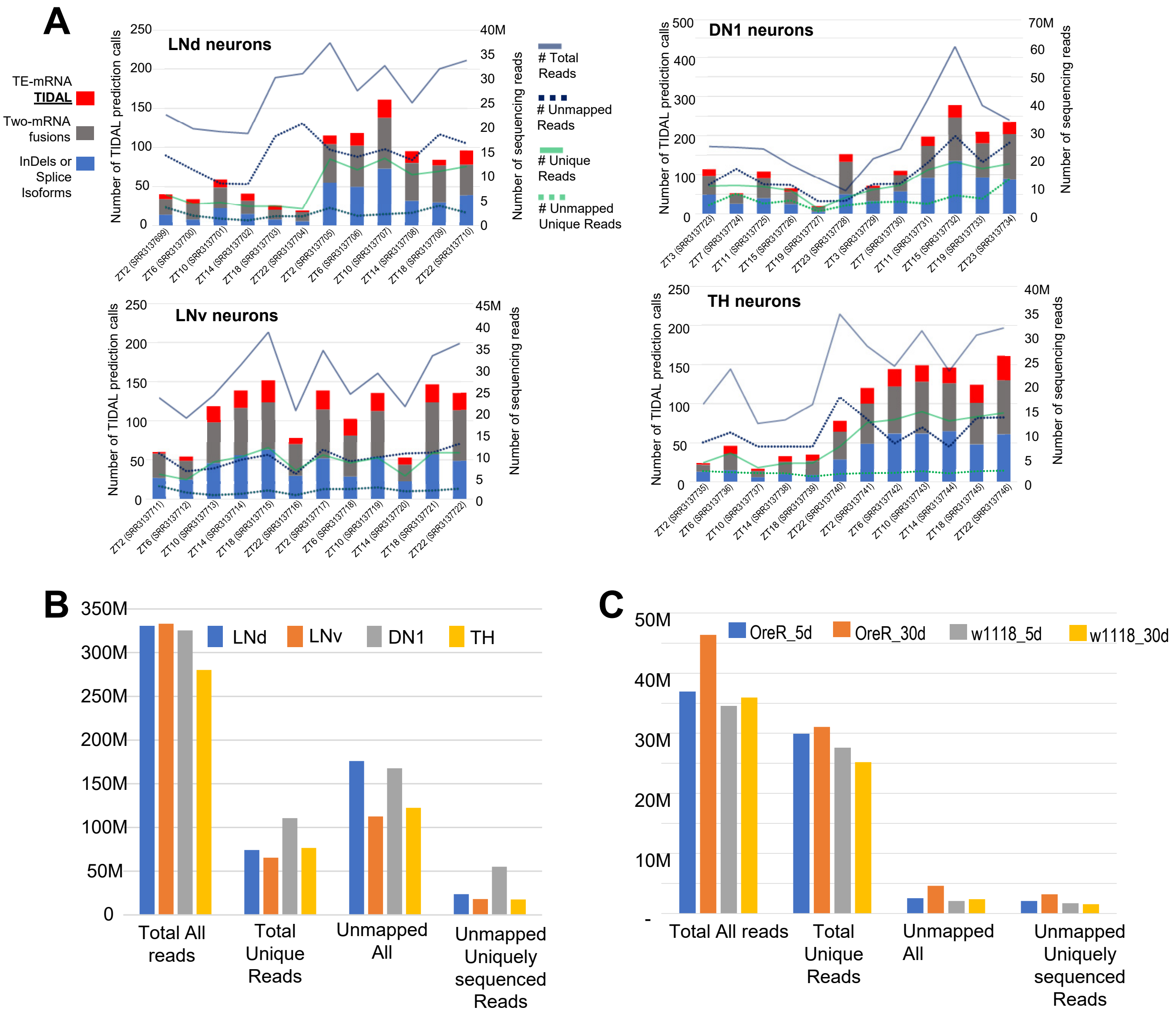

Figure S2. Supporting analyses of *Drosophila* head/brain RNAseq and DNAseq inputs into TIDAL from results in Figures 2 and 3. (A) Stacked bar and line graphs tallying the number of TE-mRNA fusion transcripts across the different ZT samples (library identifier in parentheses) in the different *Drosophila* neuronal types. TIDAL also reports deletion segments within the 100-control mRNA-coding genes and fusions between another mRNA with the control mRNA-coding gene. Note the two sets of legends corresponding to the different left and right Y-scale axis. The right Y-scale shows the number of total and uniquely-mapping reads as well as number of unmapped reads. (B) Read mapping statistics of the total RNA-seq data for each *Drosophila* neuron type were all Zeitgeiber timed samples were merged and then subjected to TIDAL analysis. (C) Reap mapping statistics of *OreR* and *w1118* wild-type whole genome DNA sequencing for comparison to RNAseq depths.
