# Supplementary material for: Transposable Element (TE) insertion predictions from RNAseq inputs and TE impact on RNA splicing and gene expression in Drosophila brain transcriptomes
Source: Mob DNA. 2024 Oct 9;15:20. doi: 10.1186/s13100-024-00330-z (PMC11462757; doi:10.1186/s13100-024-00330-z)
Supplement: Supplementary file 3 — Supplementary Material 3: Figure S3. Rigorous gDNA-PCR tests that confirm no TE insertion in the Drosophila OreR strain genome. Each figure panel is divided in parts (i) that is a diagram of the presumptive TE-gene splicing event proposed by Treiber and Waddell 2020, (ii) gel images of gDNA-PCR amplicons from the various sets of primer pairs illustrated in the diagram above the gel images. Solid lines around gels marked cropped gel images; dashed lines are demarcating lane sections on a single gel. [file 13100_2024_330_MOESM3_ESM.pdf]

Azad, Tong et al.
Figure S3.

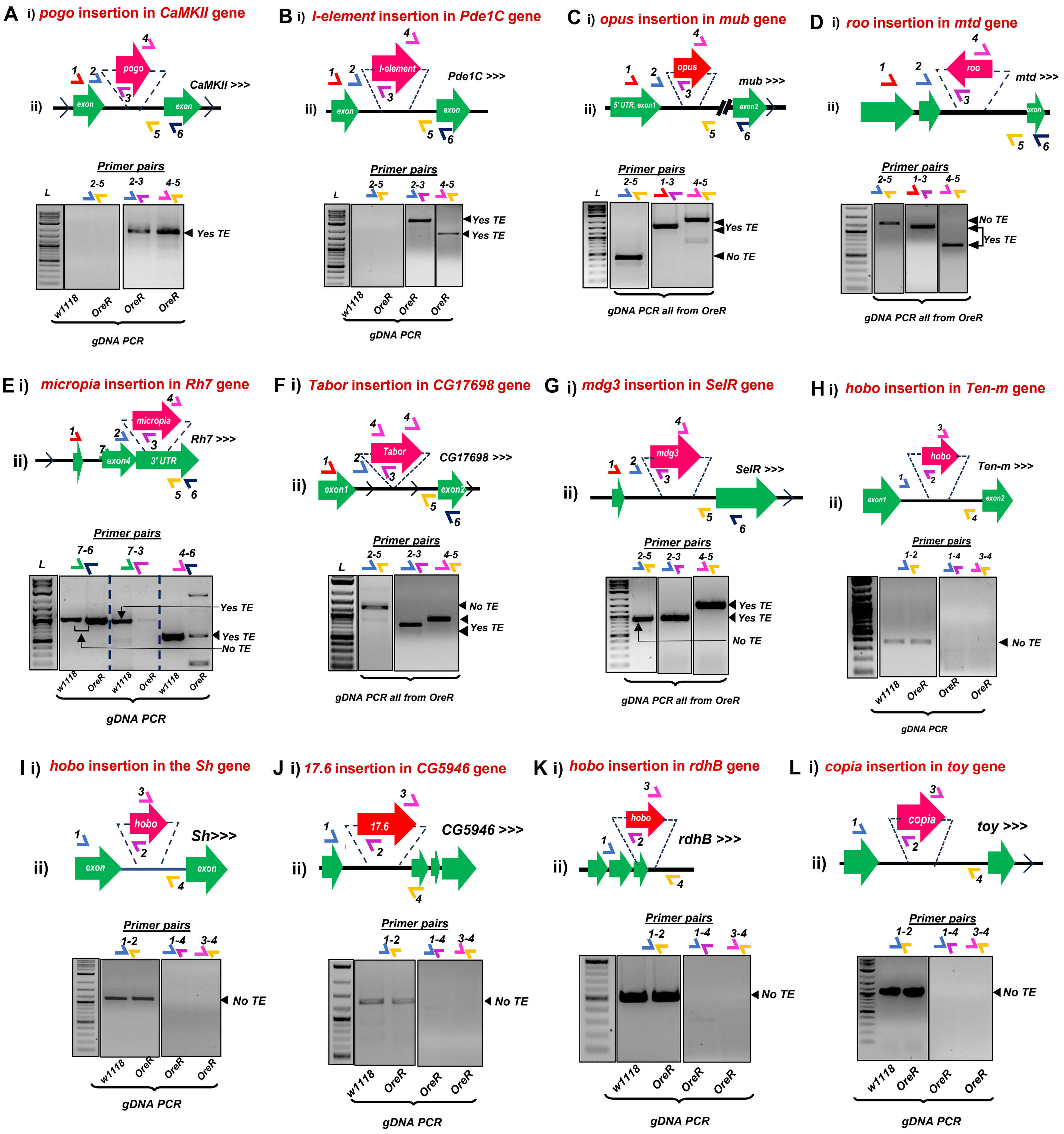

Figure S3. Rigorous gDNA-PCR tests that confirm no TE insertion in the *Drosophila OreR* strain genome.
Each figure panel is divided in parts (i) that is a diagram of the presumptive TE-gene splicing event proposed by Treiber and Waddell 2020, (ii) gel images of gDNA-PCR amplicons from the various sets of primer pairs illustrated in the diagram above the gel images. Solid lines around gels marked cropped gel images; dashed lines are demarcating lane sections on a single gel.

Azad, Tong et al.
Figure S3.

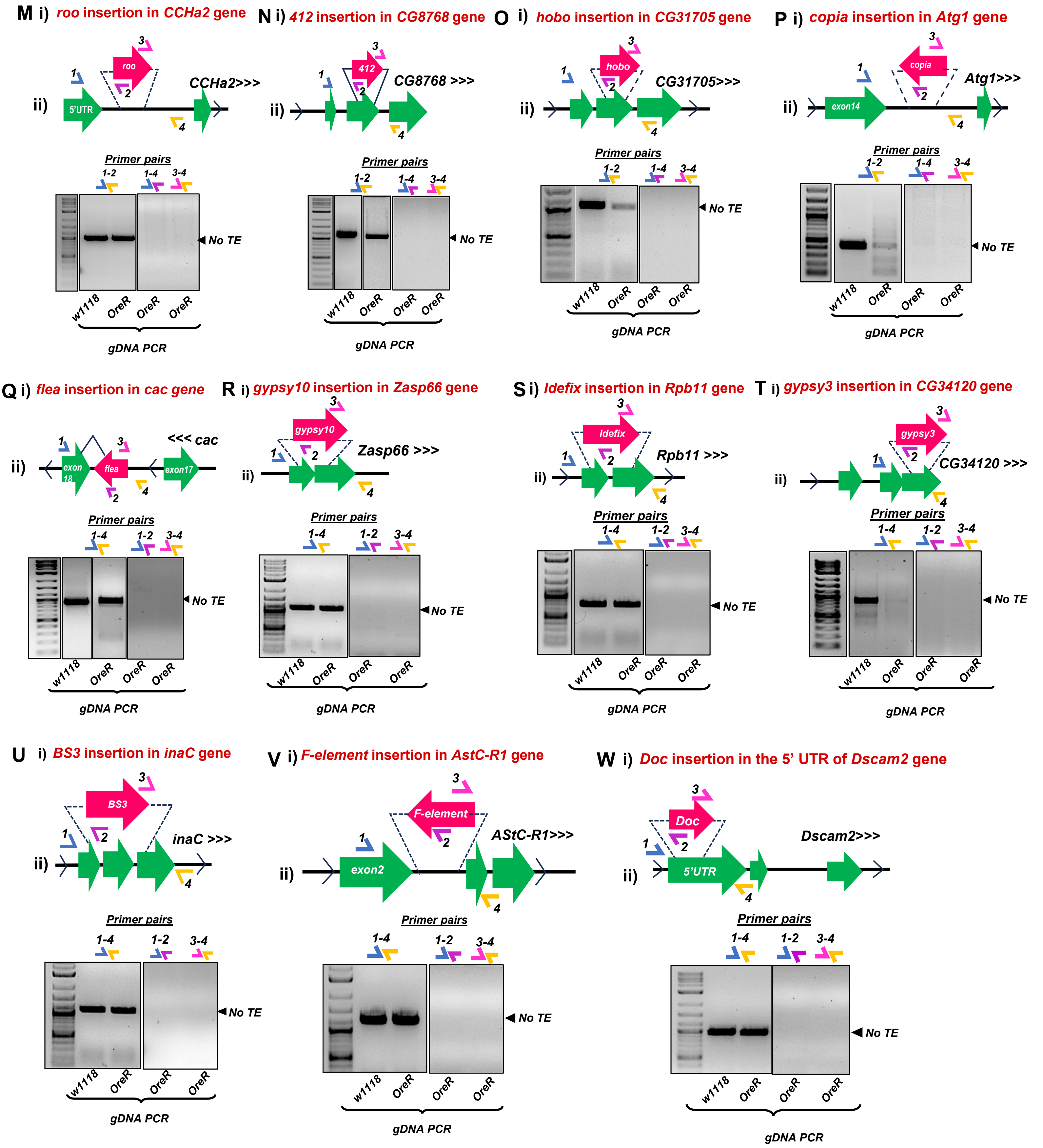

Figure S3. Rigorous gDNA-PCR tests that confirm no TE insertion in the *Drosophila OreR* strain genome.
Each figure panel is divided in parts (i) that is a diagram of the presumptive TE-gene splicing event proposed by Treiber and Waddell 2020, (ii) gel images of gDNA-PCR amplicons from the various sets of primer pairs illustrated in the diagram above the gel images. Solid lines around gels marked cropped gel images; dashed lines are demarcating lane sections on a single gel.
