# Supplementary material for: Transposable Element (TE) insertion predictions from RNAseq inputs and TE impact on RNA splicing and gene expression in Drosophila brain transcriptomes
Source: Mob DNA. 2024 Oct 9;15:20. doi: 10.1186/s13100-024-00330-z (PMC11462757; doi:10.1186/s13100-024-00330-z)
Supplement: Supplementary file 4 — Supplementary Material 4: Figure S4. WGS-DNAseq analyzed by TIDAL finds TE insertions that can exhibit signatures indicative of a “heterozygous” state, and somatic DNA copy number variation can shift and be more sporadic as flies age. (A) Box plot of the Coverage Ratio scores for all the TE insertions called by TIDAL from WGS-DNAseq libraries of w1118 flies from two adult ages, from Yang et al 2022. (B) Snapshots of the Chromosome 2L from the w1118 flies from the Yang et al 2022 study, Copy Number Variation computed by the CONTROL-FREEC program shows that as flies age, the somatic genome copy number can start to fluctuate, and these fluctuations may also contribute to the varying Coverage Ratios in measuring TE insertions from WGS-DNAseq. [file 13100_2024_330_MOESM4_ESM.pdf]

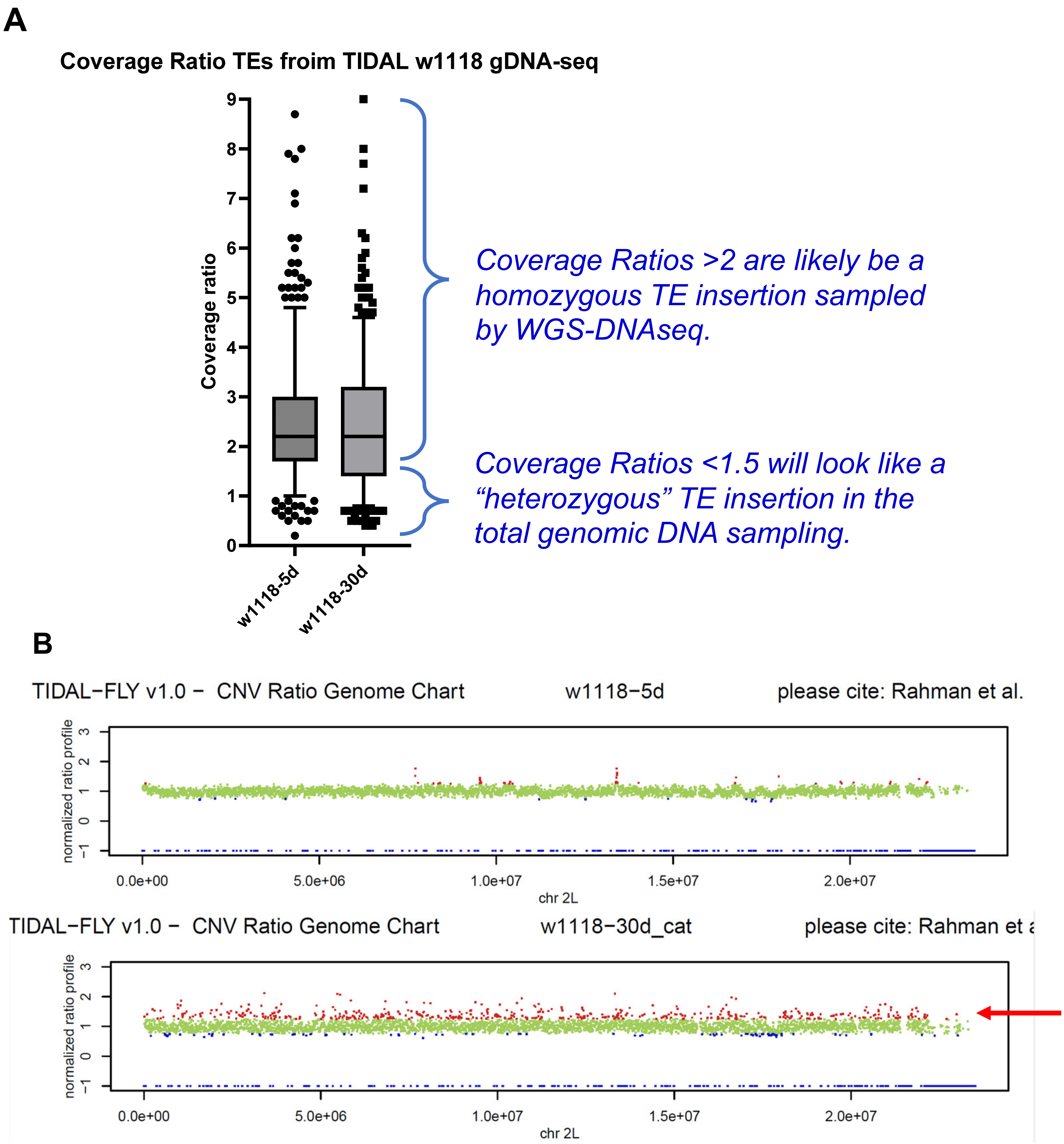


**Figure S4. WGS-DNAseq analyzed by TIDAL finds TE insertions that can exhibit signatures indicative of a "heterozygous" state, and somatic DNA copy number variation can shift and be more sporadic as flies age.** (A) Box plot of the Coverage Ratio scores for all the TE insertions called by TIDAL from WGS-DNAseq libraries of w1118 flies from two adult ages, from Yang et al 2022. (B) Snapshots of the Chromosome 2L from the w1118 flies from the Yang et al 2022 study, Copy Number Variation computed by the CONTROL-FREEC program shows that as flies age, the somatic genome copy number can start to fluctuate, and these fluctuations may also contribute to the varying Coverage Ratios in measuring TE insertions from WGS-DNAseq.
