# Supplementary material for: Transposable Element (TE) insertion predictions from RNAseq inputs and TE impact on RNA splicing and gene expression in Drosophila brain transcriptomes
Source: Mob DNA. 2024 Oct 9;15:20. doi: 10.1186/s13100-024-00330-z (PMC11462757; doi:10.1186/s13100-024-00330-z)
Supplement: Supplementary file 5 — Supplementary Material 5: Figure S5. Rigorous gDNA-PCR tests that cannot validate predicted TE insertions in the Drosophila w1118 strain genome. Panels correspond to the TE-gene pairs (A) hobo-Ten-m, (B) hobo-Sh, (C) 17.6-CG5946, (D) hobo-rdhB., (E) copia-toy, (F) roo-CCHa2, (G) 412-CG8768, (H) hobo-CG31705, (I) copia-Atg1, and (J) flea-cac. Each figure panel is divided in parts (i) that is a diagram of the presumptive TE-gene splicing event proposed by Treiber and Waddell 2020, (ii) UCSC Genome Browser snapshots of the example split reads support for the TE insertion from TIDAL analysis of the w1118 midbrain RNAseq data, (iii) gel images of gDNA-PCR amplicons from the various sets of primer pairs illustrated in the diagram above the gel images. Solid lines around gels marked cropped gel images; dashed lines are demarcating lane sections on a single gel. [file 13100_2024_330_MOESM5_ESM.pdf]

Azad, Tong et al.
Figure S5.

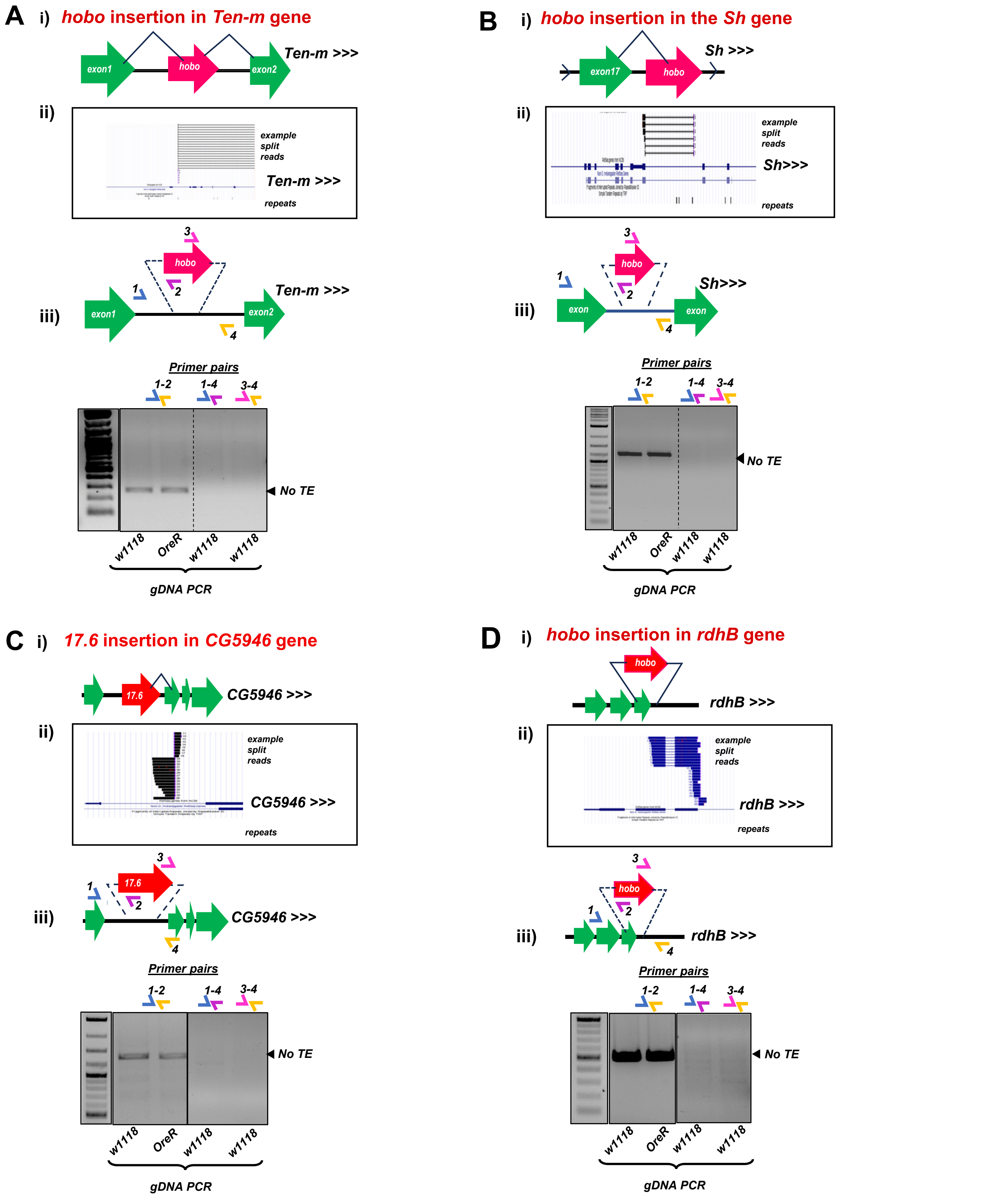

Figure S5. Rigorous gDNA-PCR tests that cannot validate predicted TE insertions in the *Drosophila w1118* strain genome. Panels correspond to the TE-gene pairs (A) *hobo-Ten-m,* (B) *hobo-Sh,* (C) *17.6-CG5946,* (D) *hobo-rdhB.***,** (E) *copia-toy,* (F) *roo-CCHa2,* (G) *412-CG8768,* (H) *hobo-CG31705,* (I) *copia-Atg1, and* (J) *flea-cac.* Each figure panel is divided in parts (i) that is a diagram of the presumptive TE-gene splicing event proposed by Treiber and Waddell 2020, (ii) UCSC Genome Browser snapshots of the example split reads support for the TE insertion from TIDAL analysis of the *w1118* midbrain RNAseq data, (iii) gel images of gDNA-PCR amplicons from the various sets of primer pairs illustrated in the diagram above the gel images. Solid lines around gels marked cropped gel images; dashed lines are demarcating lane sections on a single gel.

Azad, Tong et al.
Figure S5.

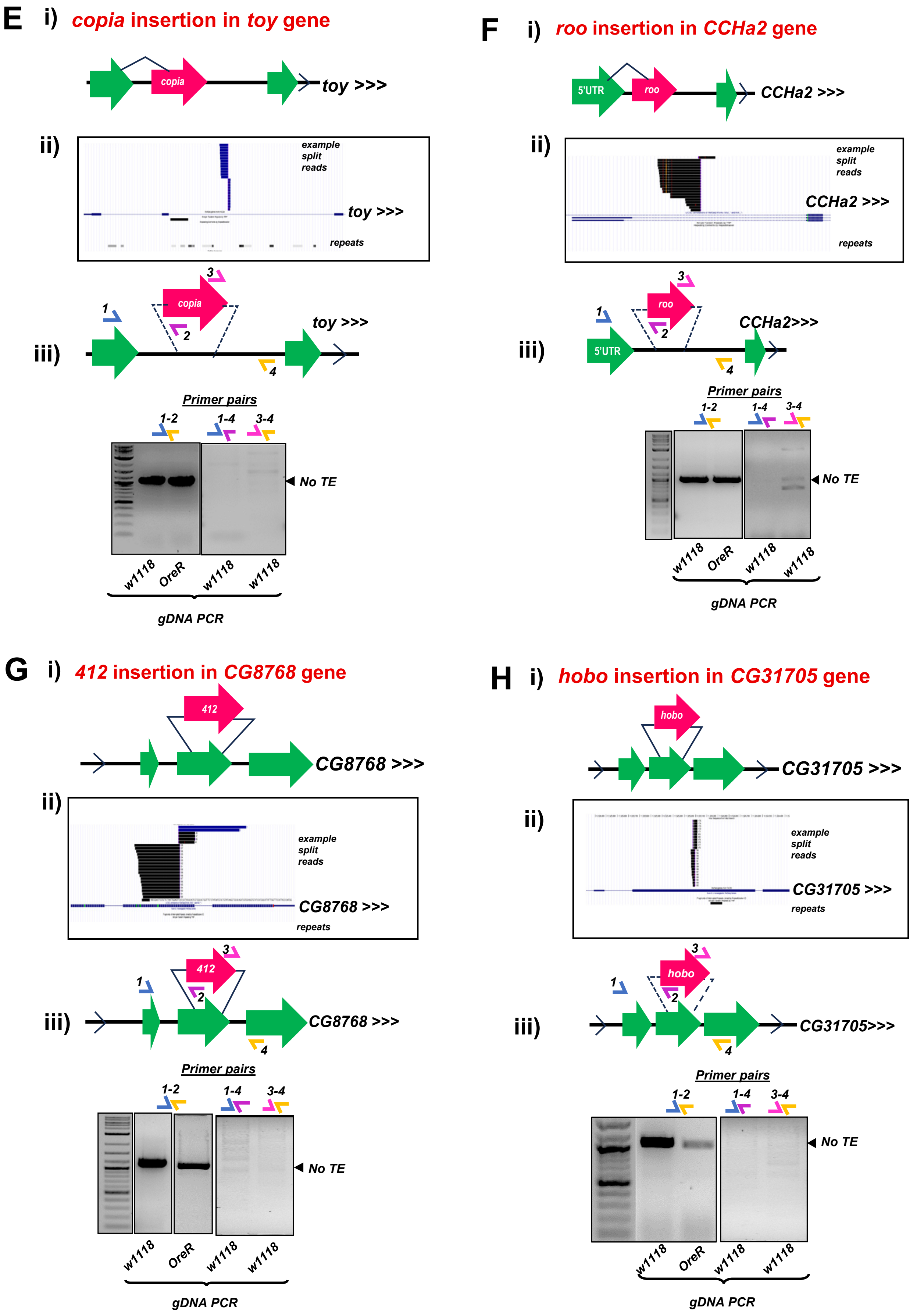

Figure S5. Rigorous gDNA-PCR tests that cannot validate predicted TE insertions in the *Drosophila w1118* strain genome. Panels correspond to the TE-gene (E) *copia-toy,* (F) *roo-CCHa2,* (G) *412-CG8768, and* (H) *hobo-CG31705.* Each figure panel is divided in parts (i) that is a diagram of the presumptive TE-gene splicing event proposed by Treiber and Waddell 2020, (ii) UCSC Genome Browser snapshots of the example split reads support for the TE insertion from TIDAL analysis of the *w1118* midbrain RNAseq data, (iii) gel images of gDNA-PCR amplicons from the various sets of primer pairs illustrated in the diagram above the gel images.

Azad, Tong et al.
Figure S5.

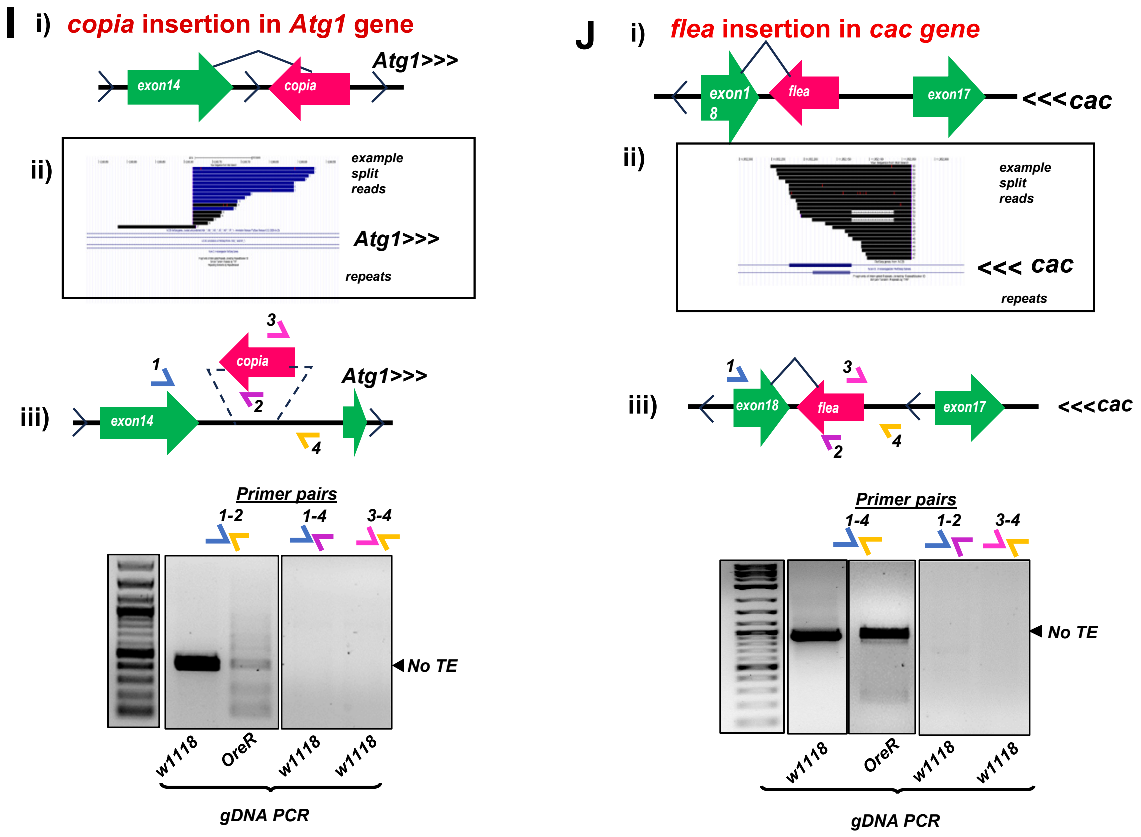

Figure S5. Rigorous gDNA-PCR tests that cannot validate predicted TE insertions in the *Drosophila w1118* strain genome. Panels correspond to the TE-gene pairs (I) *copia-Atg1 and* (J) *flea-cac.* Each figure panel is divided in parts (i) that is a diagram of the presumptive TE-gene splicing event proposed by Treiber and Waddell 2020, (ii) UCSC Genome Browser snapshots of the example split reads support for the TE insertion from TIDAL analysis of the *w1118* midbrain RNAseq data, (iii) gel images of gDNA-PCR amplicons from the various sets of primer pairs illustrated in the diagram above the gel images.
