# Supplementary material for: Transposable Element (TE) insertion predictions from RNAseq inputs and TE impact on RNA splicing and gene expression in Drosophila brain transcriptomes
Source: Mob DNA. 2024 Oct 9;15:20. doi: 10.1186/s13100-024-00330-z (PMC11462757; doi:10.1186/s13100-024-00330-z)
Supplement: Supplementary file 6 — Supplementary Material 6: Figure S6. Rigorous gDNA-PCR tests that cannot validate TE insertions predicted only by TIDAL or only by TEchim in the Drosophila w1118 strain genome. Panels correspond to the TE-gene pairs only predicted by TIDAL for (A) gypsy10-Zasp66 , (B) Idefix-Rpb11, (C) gypsy3-CG34120 , and (D) BS3-inaC Each figure panel is divided in parts (i) that is a diagram of the presumptive TE-gene splicing event (ii) UCSC Genome Browser snapshots of the example split reads support for the TE insertion from TIDAL analysis of the w1118 midbrain RNAseq data, (iii) gel images of gDNA-PCR amplicons from the various sets of primer pairs illustrated in the diagram above the gel images. Panels correspond to the TE-gene pairs only predicted by TEchim for (E) F-element-AstC-R1 , (F) Doc-Dscam2, and (G) 412-Tequila. Each figure panel is divided in parts (i) that is a diagram of the presumptive TE-gene splicing event proposed by Treiber and Waddell 2020, (ii) UCSC Genome Browser snapshots of the example split reads support for the TE insertion from TIDAL analysis of the w1118 midbrain RNAseq data, (iii) gel images of gDNA and RT-PCR (only G) amplicons from the various sets of primer pairs illustrated in the diagram above the gel images. [file 13100_2024_330_MOESM6_ESM.pdf]

Azad, Tong et al.
Figure S6.

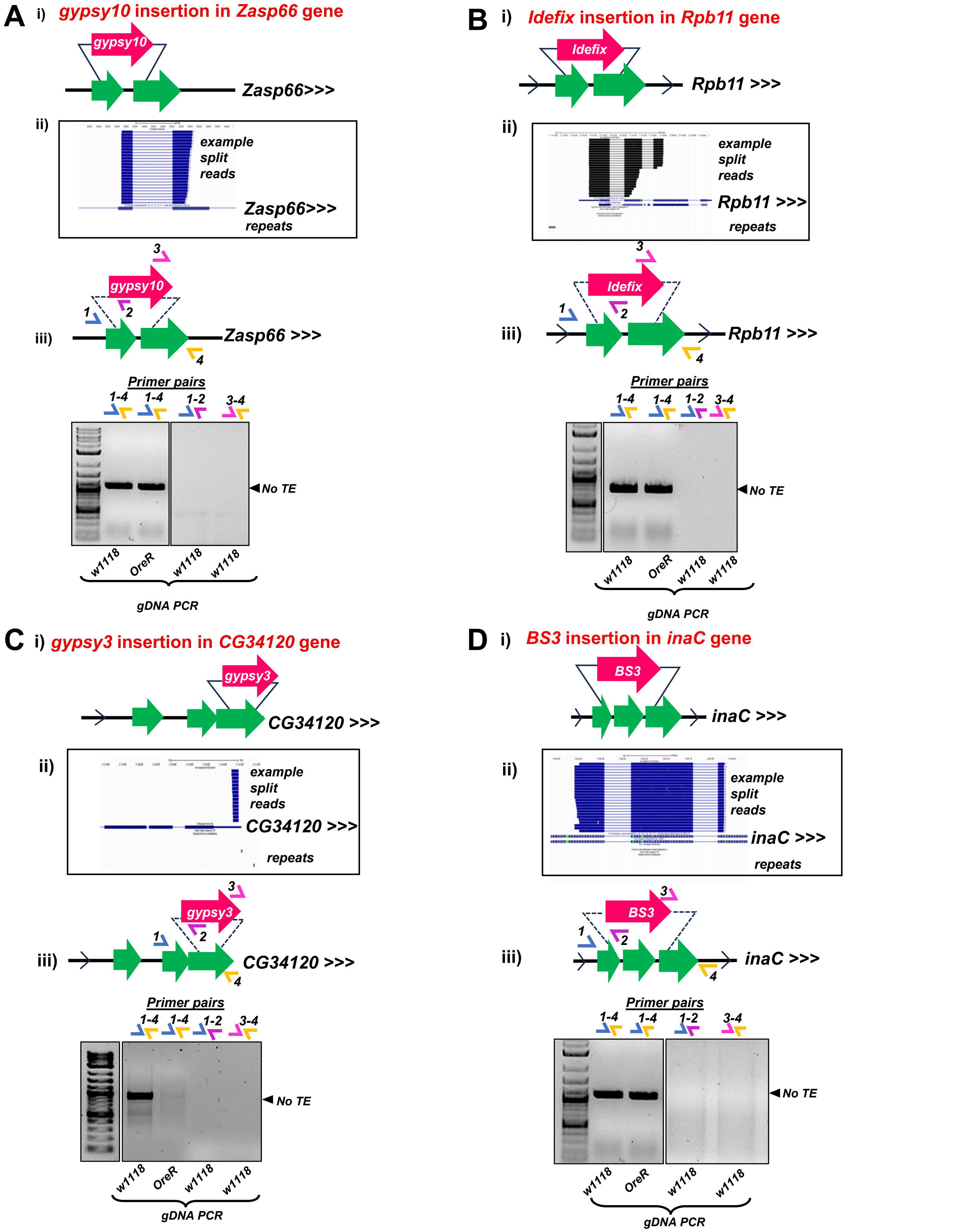

Figure S6. Rigorous gDNA-PCR tests that cannot validate TE insertions predicted only by TIDAL in the *Drosophila w1118* strain genome.

Panels correspond to the TE-gene pairs only predicted by TIDAL for (A) *gypsy10-Zasp66*, (B) *Idefix-Rpb11*, (C) *gypsy3-CG34120*, and (D) *BS3-inaC* Each figure panel is divided in parts (i) that is a diagram of the presumptive TE-gene splicing event (ii) UCSC Genome Browser snapshots of the example split reads support for the TE insertion from TIDAL analysis of the *w1118* midbrain RNAseq data, (iii) gel images of gDNA-PCR amplicons from the various sets of primer pairs illustrated in the diagram above the gel images.

Azad, Tong et al.
Figure. S6

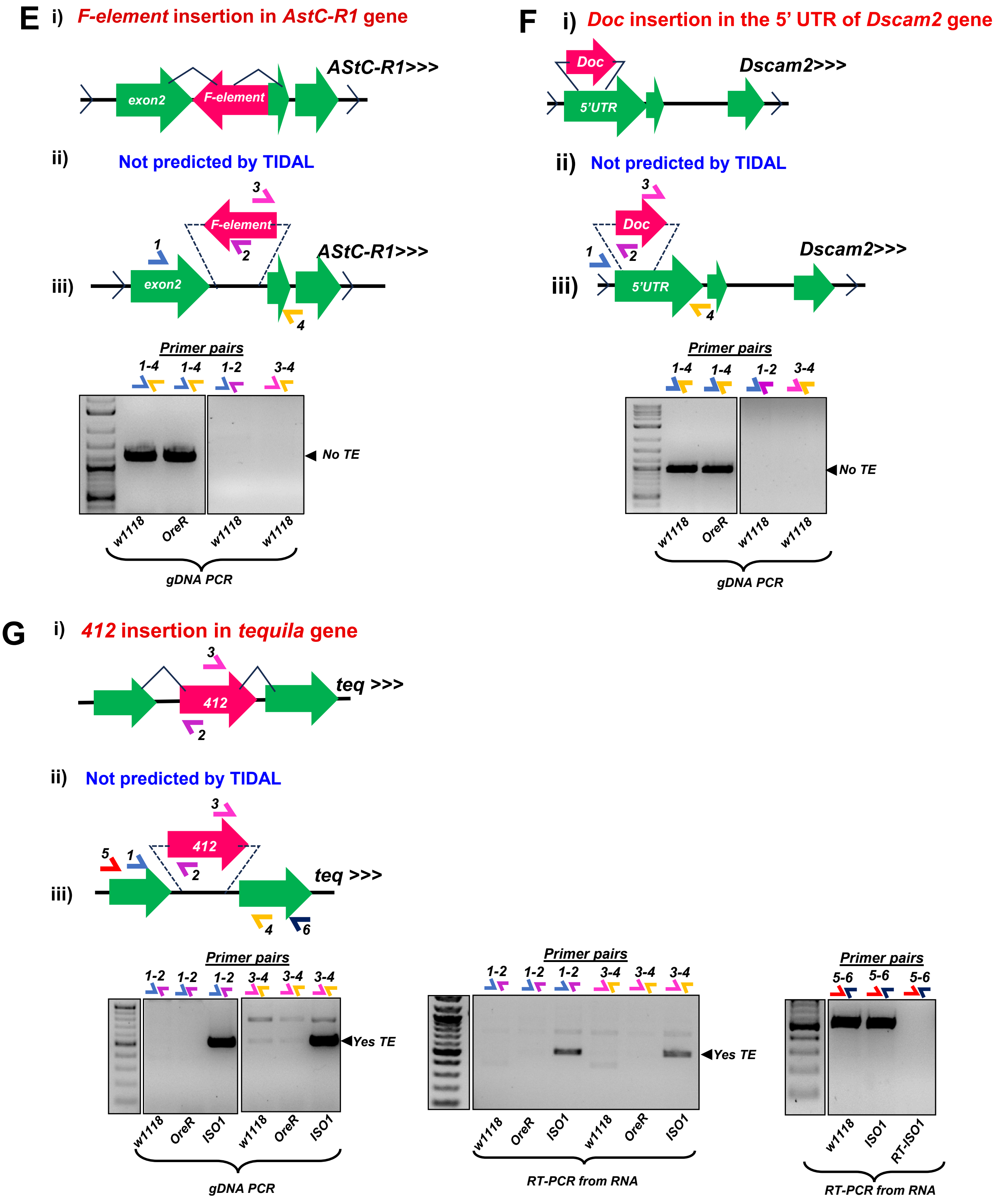

Figure S6. Rigorous gDNA-PCR tests that cannot validate TE insertions only predicted by TEchim n the *Drosophila w1118* strain genome.

Panels correspond to the TE-gene pairs only predicted by TEchim for (E) *F-element-AstC-R1*, (F) *Doc-Dscam2*, *and* (G) *412-Tequila.* Each figure panel is divided in parts (i) that is a diagram of the presumptive TE-gene splicing event proposed by Treiber and Waddell 2020, (ii) UCSC Genome Browser snapshots of the example split reads support for the TE insertion from TIDAL analysis of the *w1118* midbrain RNAseq data, (iii) gel images of gDNA and RT-PCR (only G) amplicons from the various sets of primer pairs illustrated in the diagram above the gel images.
