# Supplementary material for: Transposable Element (TE) insertion predictions from RNAseq inputs and TE impact on RNA splicing and gene expression in Drosophila brain transcriptomes
Source: Mob DNA. 2024 Oct 9;15:20. doi: 10.1186/s13100-024-00330-z (PMC11462757; doi:10.1186/s13100-024-00330-z)
Supplement: Supplementary file 7 — Supplementary Material 7: List of Gene TE pairs evaluated by PCR in this study with detailed genomic coordinates. [file 13100_2024_330_MOESM7_ESM.pdf]

Azad, Tong and Lau

TABLE S1. List of Gene TE pairs evaluated by PCR in this study with detailed genomic coordinates

		TE-Gene Pair	*TE insertion position from TIDAL*			*gPCR primer set 1 tested?* (Gene-F + Gene-R)	*gPCR primer set 2 tested?* (Gene-F + TE-R)	*gPCR primer set 3 tested?* (Gene-R + TE-F)	*Forward primer position*	*Reversed primer position*	*Expected size*
Gene-TE pairs predicted in a *w1118* strain derivative by Treiber & Waddell	1	*blood-Dscam2*	chr3L	7183892	7184420	√	√	√	7184411 7184431	7183660 7183682	750bp
	2	*opus-Bx*	chrX	18550430	18550670	√	√	√	18549894 18549914	18551094 18551115	1221bp
	3	*I-element-Pde1C*	chr2L	11860287	11860520	√	√	√	11860203 11860222	11860793 11860816	613bp
	4	*pogo-CaMKII*	chr4	1038286	1038654	√	√	√	1037963 1037983	1038768 1038787	824bp
	5	*copia-toy*	chr4	993551	993832	√	√	√	993165 993184	994255 994274	1100bp
	6	*roo-CCHa2*	chr3R	13392387	13392665	√	√	√	13392841 13392860	13391900 13391921	950bp
	7	*hobo-CG31705*	chr2L	11525040	11525499	√	√	√	11525733 11525754	11524726 11524748	1028bp
	8	*flea-cacophony*	chrX	11951970	11952244	√	√	√	11952372 11952391	11950263 11950282	2183bp
	9	*17.6-CG5946*	chr3L	11819104	11819395	√	√	√	11818826 11818847	11819706 11819724	898bp
	10	*hobo-rdhB*	chr3R	22352597	22352904	√	√	√	22352446 22352464	22353113 22353135	689bp
	11	hobo-Ten-m	chr3L	22356882	22357046	√	√	√	22356884 22356904	22357114 22357133	250bp
	12	*hobo-Sh*	chrX	17949931	17950074	√	√	√	17947486 17947508	17946730 17946749	778bp
	13	412-CG8768	chr2R	12691622	12692103	√	√	√	12692579 12692598	12691455 12691475	1143bp
	14	*Tabor-CG17698*	chr3L	23742885	23743145	√	√	√	23740772 23740792	23743295 23743314	700bp
	15	*opus-mub*	chr3L	21845047	21845208	√	√	√	21844858 21844877	21845300 21845319	461bp
	16	*roo-mtd*	chr3R	5284197	5284467	√	√	√	5285131 5285153	5283896 5283916	1257bp
	17	*micropia-Rh7*	chr3L	12165142	12165476	√	√	√	12165546 12165568	12164902 12164922	666bp
	18	copia-Atg1	chr3L	12800567	12800901	√	√	√	12800410 12800430	12801004 12801025	615bp
	19	mdg3-SelR	chr3R	10865047	10865455	√	√	√	10865954 10865973	10865061 10865080	912bp
	20	*Doc-Dscam2*	Not predicted by TIDAL			√	√	√	89904 7189924(5'UT	7188926 7188947(intron)	924bp
	21	*F-element-AstC-R1*	Not predicted by TIDAL			√	√	√	11077596 11077616	11076383 11076403	1233bp
	22	*412-Tequila*	Not predicted by TIDAL			√	√	√	9076674 9076694	9084647 9084666	7992
Gene-TE pairs predicted in the w1118	23	*gypsy3-CG34120*	chrX	21140649	21140805	√	√	√	21139880 21139899	21140880 21140901	1021bp
	24	*gypsy10-Zasp66*	chr3L	8634596	8634742	√	√	√	8634080 8634100	8635117 8635140	1060bp
	25	*BS3-inaC*	chr2R	16901112	16901335	√	√	√	16900275 16900294	16901335 16901355	1080bp
	26	*Idefix-Rpb11*	chr2L	17409980	17410152	√	√	√	17410447 17410466	17409609 17409628	838bp
