# Supplementary material for: Transposable Element (TE) insertion predictions from RNAseq inputs and TE impact on RNA splicing and gene expression in Drosophila brain transcriptomes
Source: Mob DNA. 2024 Oct 9;15:20. doi: 10.1186/s13100-024-00330-z (PMC11462757; doi:10.1186/s13100-024-00330-z)
Supplement: Supplementary file 8 — Supplementary Material 8: List of oligonucleotides used in this study. [file 13100_2024_330_MOESM8_ESM.pdf]

Azad, Tong and Lau

Table S2. List of oligonucleotides used in this study

**genomic PCR Primer list**	
Dm_Dscam2_Doc-Fw	CTGTTCGAGGCTCGAAGGATA
Dm_Dscam2_Doc-Rv	ATTCTTGGCCGACTCTTTTCTC
Dm_Doc-Internal-Fw	CACATTGTCGCTGAGAACGTAT
Dm_Doc-Internal-Rv	CATATCTGGGAGTTCATCTGGA
Dm_AstC-Fw	ACAGCAATCTGCGTCAGCAAG
Dm_AstC-Rv	TACCACACCATATGCAAAGCG
Dm_CCHa2-Fw	AGCCCAGTTCGGCTAATCAG
Dm_CCHa2-Rv	GTGCAGATAACGACCAGTAGCA
Dm_roo-LTR-Fw	AGGTGACATGAGAATCGCATC
Dm_roo-LTR-Rv	GTGCACACTACATGAGTCAGTC
Dm_toy-Fw	AGGCAAAAACGCAGCGTAAG
Dm_toy-Rv	GTTGGTGTAAGAAGGGGCGT
Dm-Copia-Fw	GTGAGTAGGTCGTGGTGCTG
Dm-Copia-Rv	ACCAGCACCACGACCTACTC
Dm_Bx-Fw	TGAGGACGATACAGCACACAC
Dm_Bx-Rv	ACTTGCATCAGGGTTCTCGG
Dm_opus-Fw	CCATCGCTCTTACGGTCCAA
Dm_opus-Rv	ACGGCACTTGTTCCGAATGC
Dm_Rpb11-Fw	AGCTAGGCGTTCTAGCTACT
Dm_Rpb11-Rv	TATTGAATGCGATTCGTGGC
Dm_CG34120-Fw	TCTCCGAACTCTTCGCCATC
Dm_CG34120-Rv	CTTCAGACAGATCCCATTAGGA
Dm_inaC-Fw	ATGAGCTGTATGCCGTGAAG
Dm_inaC-Rv	TCCGTTGGAGTTAAGTCCGTC
Dm_gypsy3-Fw	ACTAAGTTAACCGGACTGATCGTC
Dm_gypsy3-Rv	GAGCCATCGCTCATTAACCAACA
Dm_Zasp66-Fw	ACGAGCAACAGCTGATCAAGC
Dm_Zasp66-Rv	CATTGTATCGAGCTAAGCATAGGA
Dm_CG5946-Fw	CTGCTGAATAAGAAGTCCACGA
Dm_CG5946-Rv	TAGCCATGACGTCACAGCA
Dm_17-6-Fw	AGCGGCACTTAGCCATTCTT
Dm_17-6-Rv	AGGAGAAGCCTCTGTGCTTG
Dm_CG17698-Fw	TCAATCGATATGCTAACAAGAACGC
Dm_CG17698-Rv	ACGGACATGTACAGATCGACTC
Dm_Tabor-LTR-Fw	ATTCAGACCAGAAGTGCAGAGTC
Dm_Tabor-LTR-Rv	GAAGGCTCTTTGACGACTCCT
Dm_CaMKII-Fw	CTGACATGATCGACTCAGCTA

Dm_CaMKII-Rv	TCACTAGCCGAGTCGACTTG
Dm_pogo-Fw	TGCATCGATAGTTAGCTGCATC
Dm_pogo-Rv	GCTTAGCTGCCTCGAGTACT
Dm_rdhB-Fw	AGCTCGTCGAGTGGAACTC
Dm_rdhB-Rv	GAACACTTTCTACGGAATTGGCT
Dm_Ten-m-Fw	AGTTCTGTAAGCGCAGATAAGAC
Dm_Ten-m-Rv	CTGTCCTTAAAGGATTTCGCAG
Dm_Sh-Fw	ACTGCTACTTATGAACCGTAACG
Dm_Sh-Rv	TGAATTGCGATCCGAATGCG
Dm_hoboFw	ATCGTTGACTGTGCGTCCACT
Dm_hobo-Rv	TGCGCACCCGAATCAATACG
Dm_Pde1c-Fw	CCGAAGCCATATGGCAATGA
Dm_Pde1c-Rv	GTCTAACTATAGCTGTACAGACCA
Dm_I-element-Fw	ACGCTGGATAGGAGTTGAGATG
Dm_I-element-Rv	TGAGAGGCACGACTTATCTCTTC
Dm_Rh7-Fw	AGACCGAACTGAAATCAGCAATG
Dm_Rh7-Rv	ACCACCGGATTAGACATCGAG
Dm_micropia-Fw	ATCGTGGAGAAGCCAAAGCA
Dm_micropia-Rv	GTCCGTCCGTCCATATCAGC
Dm_Atg1-Fw	TAGGTGCAATGGCCTCTCAAC
Dm_Atg1-Rv	ACAGCGTAAATACACGGAAGTC
Dm_mub-Fw	ATCAGCCGACCGAGTTTGAC
Dm_mub-Rv	ATGGCAACAAGAACTGCTCG
Dm_opus-Fw	CACTCAGGGTGAGGGGTCAA
Dm_opus-Rv	TCGAGACTGGGACCTCTTCT
Dm_CG8768-Fw	AGGCATGCTCTGATAGGTAC
Dm_CG8768-Rv	TGCGCTTGGTACTAACATCTA
Dm_SelR-Fw	TGTTCCACTGCCCAGAGTTC
Dm_SelR-Rv	CCAGGAACTGTAGACTCGCA
Dm_mdg3-Fw	TAATCGACTCGCCACTCTGC
Dm_mdg3-Rv	TAGCCGCCGTTTACAGAAGT
Dm_mtd-Fw	TGCACGTATGCTCTCGTATCAAC
Dm_mtd-Rv	AGCATTCCAACTCGAAGTTGC
Dm_roo-LTR-Fw	AGGTGACATGAGAATCGCATC
Dm_roo-LTR-Rv	GTGCACACTACATGAGTCAGTC
Dm_cac-Fw	ATAGAGTTTTAGCCATGCAACTC
Dm_cac-Rv	TGAGCTAGGCTATTAGTAGTTTGA
Dm_flea-LTR-Fw	AGTGGAAGTCAGCGTTGCAG
Dm_flea-LTR-Rv	CGACAATCATGTTGCTGCTCA
Dm_CG31705-Fw	ACATATGTATGTGGCAGGCATG
Dm_CG31705-Rv	ACTGGATGGTAAGATGCTCCTGC
Dm-Teq-Rv	AGGTGGGCAGGAAATGTCAC
Dm-Teq-Fw	AGAGATCATATGCTGTCCCAC

Dm-412-Rv	ATTTGGTCGGCGTGTGAATG
Dm-412-Fw	AAGTGCATTGCCCACTCGAA
Dm_BloodLTR-Fw	CTAAGTCAGCATCCCCACGC
Dm_BloodLTR-Rv	CTGTAATAAACCAATATATGCC
Dm_Dscam2-Fw	ACTTTCGAATGCGACAATGGC
Dm_Dscam2-_Rv	CACCGAATTGCTGGCAATACAGC
Dm-blood-cds3-FW1	ATGCGAATATCACCAAGCGAAC
Dm-blood-cds3-FW2	CTATAGAAGGAGGCCACACTG
Dm-blood-cds3-FW3	CCAGATGACCAAGACAATCTCA
**qRT-PCR and ddPCR primer list**	
Dm_Dscam2-Ex1-Fw	ATTGTACAGCTCACTGCCCAC
Dm_Dscam2-Ex2-Rv	GCTTCGAACGGTTCACTGCA
Dm_Dscam2-Ex3-Fw	AGGAATTGGTACGAGTGGTCTC
Dm_Dscam2-Ex4-Rv	AGATTGTGGATGAGCAGCTC
Dm_Dscam2-Ex4-Fw	AGAGCGATGAGTCGCAGTC
Dm_Dscam2-Ex5-Rv	GTATTCGGGCGAAGGACAG
Dm_Bx-Ex1-Fw	GCAACAGTAAGAGCCTTGTGC
Dm_Bx-Ex2-Rv	ACCGGGTTCGGTTTTGGTAA
Dm_Bx-Ex2-Fw	AGCCTTCGAGATGGTGATGC
Dm_Bx-Ex3-Rv	ATTGGCCATCGATGCGAAGAC
**RT PCR Primer List**	
Dm_mtd-UTR-Fw1	TCTCCACGATTCTGCGATTCTTC
Dm_mtd-Exon3-Rv1	TGTTGTGAGGTGCTGAAGATCAG
Dm_mub-UTR-Fw1	AGTGCATAATCCTTCAACAGC
Dm_mub-exon-Rv1	ATCCTCGTGTTTGATGGACG
Dm_Rh7-UTR-Fw1	AGACCGAACTGAAATCAGCAATG
Dm_Rh7-UTR-Rv1	ACCACCGGATTAGACATCGAG
Dm_Pde1C-UTR-Fw1	TGGAAGCCATTGCAGCTAAC
Dm_Pde1C-exon-Rv1	TAGTTGGTTGCATTCGGCGATG
Dm_SelR-Exon-Fw1	ACCAGGATCTGTTCAGCTCG
Dm_SelR-Exon-Rv1	GAATTGATGCAGTAGCGCTTGC
Dm_CaMKII-exon-Fw1	AGGAACCGTTGATCGCAGTAC
Dm_CaMKII-exon-Rv1	TCTACAAGGTTACCCAGTGCTTC
Dm_CG17698-FW1	ACAAGTCCTAAGGAAAGAAATGC
Dm_CG17698-RV1	ATGAGTGGAATCTTCTTCGGAG
